# Investigation of oxidative characteristics, fatty acid composition and bioactive compounds content in cold pressed oils of sunflower grown in Serbia and Argentina

**DOI:** 10.1016/j.heliyon.2023.e18201

**Published:** 2023-07-15

**Authors:** Tanja Lužaić, Snežana Kravić, Zorica Stojanović, Nada Grahovac, Siniša Jocić, Sandra Cvejić, Lato Pezo, Ranko Romanić

**Affiliations:** a Faculty of Technology Novi Sad, University of Novi Sad, Bulevar Cara Lazara 1, 21000 Novi Sad, Serbia; b Institute of Field and Vegetable Crops, National Institute of the Republic of Serbia, Maksima Gorkog 30, 21000 Novi Sad, Serbia; c Institute of General and Physical Chemistry, University of Belgrade, Studentski trg 12/V, 11000 Belgrade, Serbia

**Keywords:** Cold pressed oil, Totox index, Oxidative stability prediction, Artificial neural network

## Abstract

**Background:**

In this work, the chemical composition analysis was performed for cold pressed oils obtained from the 15 sunflower hybrids grown in Serbia and Argentina, as well as the determination of their oxidative quality. The fatty acid composition and bioactive compounds including total tocopherols, phenols, carotenoids, and chlorophyll contents were investigated. The oxidation products were monitored through the peroxide value (PV), anisidine value (AnV), conjugated dienes (CD) and conjugated trienes (CT) content, and total oxidation index (TOTOX) under accelerated oxidation conditions by the oven method.

**Results:**

Linoleic acid was the most abundant fatty acid in investigated oil samples, followed by oleic and palmitic acids. The mean contents of total tocopherols, phenols, carotenoids, and chlorophyll were 518.24, 9.42, 7.54 and 0.99 mg/kg, respectively. In order to obtain an overview of sample variations according to the tested parameters Principal Component Analysis (PCA) was applied.

**Conclusion:**

PCA indicated that phenols, chlorophyll, linoleic and oleic acid were the most effective variables for the differentiation of sunflower hybrids grown in Serbia and Argentina. Furthermore, based on the fatty acid composition and bioactive compounds content in the oils, a new Artificial Neural Network (ANN) model was developed to predict the oxidative stability parameters of cold pressed sunflower oil.

## Highlights

• Sunflower seed cold pressed oils (CPOs) were investigated.
• CPOs are produced from seeds of newly created hybrids grown in Serbia and Argentina.
• CPO significant difference in C18:1 and C18:2 fatty acids content was found.
• CPOs from Argentina had higher total phenols and chlorophyll content.
• ANN model was developed to predict oil oxidative characteristics.

## Graphical abstract

**Figure:**
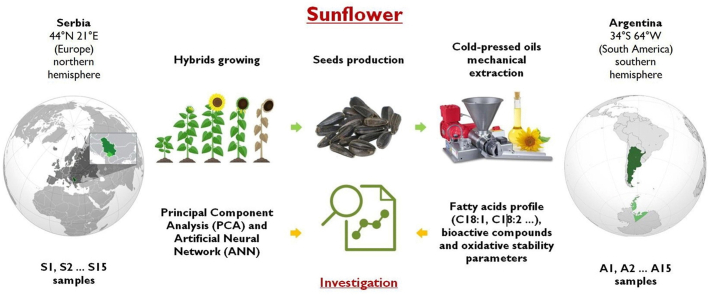

## 1 Introduction

Oxidative stability is one of the most important parameters that affects the oil quality and it is closely related to the fatty acids compositions of oils, as well as some minor antioxidant components and numerous external factors [1,2]. The fatty acids composition of sunflower oil is greatly influenced by the location and climatic conditions during vegetation [3,4]. Higher temperatures during the period of seed development and oil synthesis contribute to the oleic acid content increases and the linoleic acid content decreases [[5], [6], [7]]. Besides, high differences between day and night temperatures result in an increase in linoleic acid accumulation [7]. In sunflower seed oil linoleic and oleic fatty acids constitute approximately 90% of the total fatty acids present [8,9]. Higher rate of unsaturated and polyunsaturated fatty acids such as linoleic and linolenic acids promote oxidation process. The presence of double bounds in these fatty acids can offer several possibilities to carrying out of the chemical modification of the structure to improve some of their properties [10]. Fatty acids in a non-radical singlet state do not react directly with atmospheric oxygen [1]. In the initiation phase, hydrogen atoms are being removed to form alkyl radicals, which further react with atmospheric oxygen to form a peroxyl radical. Hydroperoxide and oxy radical are being formed in the propagation phase, while in the termination phase pentanes are being formed [11]. For the formation of free radicals on the C8 atom of oleic acid and C11 atom of linoleic acid, 75 kcal/mol and 50 kcal/mol, respectively, are required. The oxidative rates of oleic acid, linoleic acid and linolenic acid are 1:12:25 [12]. Autoxidation of edible oils and fats can also be catalyzed by other factors such as exposure to light, heat, and transitional metals. This process is a free radical chain reaction, leading to increase in reactive radicals, which initiate further reactions [13,14]. As a consequence, unfavorable changes may happen, including deterioration in the sensory properties, decrease in nutritional value, and occurrence of chemical compounds harmful to the human health [[15], [16], [17]]. Oxidative changes in oil can be monitored through primary and secondary oxidation products. These oxidation products are indicated by peroxide value (PV), anisidine value (AnV), conjugated dienes (CD) and conjugated trienes (CT) content [18].

Minor compounds present in the oil also have an effect on oxidative stability. The majority of minor compounds, including tocopherols, phenols, carotenoids and chlorophyll derivates, show a bioactive effect. Tocopherols act as scavengers of free radicals and their biological value differs depending on the isomer. α-Tocopherol has the highest biological activity with antioxidant and anti-inflammatory properties in human body, while γ-tocopherol has the highest antioxidant activity [19]. In all, primary role of tocopherols is the prevention of the oxidation of polyunsaturated fatty acids by membrane phospholipids. Natural tocopherols are present in sunflower oil in four isomers: α- (5,7,8-trimethyltocol), β- (5,8-dimethyltocol), γ- (7,8-dimethyltocol), and δ- (8-methyltocol). Standard sunflower oil contains mostly α-tocopherol (95%), and lower quantities of β-tocopherol (3%) and γ-tocopherol (2%) [20,21]. 91% of total tocopherols in sunflower oil are α-tocopherol and range from 403 to 935 mg/kg, while the content of total tocopherols in sunflower oil ranges from 440 to 1520 mg/kg [19,22]. According to Gotor et al. [23], Marmesat et al. [24], and Velasco et al. [25] the content of total tocopherols ranged from 176.9 to 1872 mg/kg for standard linoleic sunflower seed oil, 450–1120 mg/kg for high oleic sunflower oil and 509–741 mg/kg for mid oleic sunflower oil.

Phenols have a strong antioxidant effect. These compounds act by interrupting the oxidation chain reaction by donating hydrogen to free radicals formed in the oxidation process, resulting in stable antioxidant radicals. Sunflower oil is not rich in phenols, but sunflower seed contain significant amount of phenolic compounds (1–3 g of phenols per 100 g of seeds) [26]. After the oil extraction, the phenolic compounds generally remain in the cake. Of the phenolic compounds in sunflower seed, as well as in the sunflower oil, the most common is chlorogenic acid, while cinnamic, coumaric, ferulic, sinapic and hydroxy-cinnamic acids are found in small amounts and vanillic, syringic and hydroxy-benzoic acids in trace amounts [[27], [28], [29]]. Despite the minor content of phenolic compounds in sunflower oil, their positive impact on the oil quality and stability is significant [30,31].

Carotenoids play the role of free radical scavengers in both in vitro and in vivo [[32], [33], [34]]. Those substances are 40-carbon isoprenoids or tetraterpenes with different structural characteristics. Also, carotenoids are valuable micronutrients with strong biological and protective function in the body [[35], [36], [37]]. β-carotene has the ability to interact with free radicals, including peroxyl radicals [38,39]. The total carotenoids content in cold pressed sunflower oil ranges from 2 to 4 mg/kg [37]. Dimić et al. [40] found significantly higher total carotenoids content in cold pressed sunflower oil (from 4.80 to 14.43 mg/kg). Other cold pressed oils usually are containing less carotenoids. Walnut oil contains 0.93 ± 0.05 mg/kg [41], grape seed oil 0.2 mg/kg [42] and blueberry oil 19 μg/kg [43].

Chlorophyll and chlorophyll derivatives are the most active promoters of oil photooxidation in the presence of light and largely make oil susceptible to oxidative processes. According to some authors, chlorophyll derivatives, pheophytin A, showed a mild antioxidant effect in the dark, probably by donating hydrogen to free radicals, thus interrupting the oxidation chain reaction [44].

Phytosterols are important nutritional components of vegetable oils. Structurally phytosterols are similar to cholesterol, so in addition to lowering blood cholesterol levels, some studies indicate that phytosterols have anticancer, antiatherosclerosis, anti-inflammatory and antioxidant properties [45]. From a technological aspect, phytosterols increase the thermal stability of oils with a high content of polyunsaturated fatty acids [46]. The most abundant phytosterols in standard sunflower oil are β-sitosterol (60% of total desmethylsterols), Δ7-stigmasterol (14%), campesterol (8%) and stigmasterol (8%). Vlahakis and Hazebroek [47] found maximum concentrations of 20.4% campesterol, 17.9% stigmasterol and 81.5% β-sitosterol.

In this study, the oxidative characteristics of cold pressed oils obtained from the same sunflower seed grown in Serbia and Argentina were investigated. The fatty acids composition, as well as the content of the bioactive compounds (total tocopherols, phenols, carotenoids, and chlorophyll) present in the oil, affecting the oxidative stability, were examined. Namely, oxidation products were monitored through the PV and AnV, as well as CD and CT content under accelerated oxidation conditions by oven method. The main goal of this paper was to differentiate oil samples grown on the territory of Serbia and Argentina according to the fatty acids profile, bioactive compounds and oxidative stability parameters by application of Principal Component Analysis (PCA). Additionally, Artificial Neural Network (ANN) was applied in order to test the capability to predict the stability parameters according to the fatty acid composition and bioactive compounds content in the cold pressed sunflower oils.

## 2 Material and methods

### 2.1 Samples and sample preparation

Fifteen sunflower hybrids (standard linoleic-type) developed at the Institute of Field and Vegetable Crops Novi Sad and commercially used in Serbia, Ukraine, Russia and some EU countries were used in this study - Dusko, Orfej, NS Oskar, NS Konstantin, NS Samuraj CLP, Pegaz, NS Smaragd CLP, NS Romeo, NS Kruna, NS Fantazija, Sumo 1 PR, Sumo 2OR, NS Sumo Sjaj, NS Sumo Sol, NS Ronin. During the 2017 growing season, study was carried out in two locations in two representative latitudes: Novi Sad, Serbia at the experimental field of the Institute of Field and Vegetable Crops in north (45°15′6.01″N, 19°50′12.98″E) and Pergamino, Argentina in south (33°53′23.82″S, 60°34′24.85″W) at the experimental field of Asociación de Coop Argentinas C.L. (ACA). Fifteen hybrids grown in Serbia were marked as S1, S2, S3, S4, S5, S6, S7, S8, S9, S10, S11, S12, S13, S14 and S15, while the same fifteen hybrids grown in Argentina were marked as A1, A2, A3, A4, A5, A6, A7, A8, A9, A10, A11, A12, A13, A14 and A15. Argentina, as the largest sunflower seed producer in the Southern Hemisphere, was chosen for the comparison. The adaptability of the examined hybrids growing conditions on the territory of Argentina was investigated, through minor components and oxidative stability. Growth conditions, seed preparation for cold pressing, as well as cold pressing conditions and obtained oil treatment were described by Lužaić et al. [48] and Lužaić et al. [49] This oil extraction method was chosen because of the minimal impact on the fatty acids composition and the bioactive compounds content.

### 2.2 Fatty acid composition

The fatty acid compositions of cold pressed sunflower oils were determined by gas chromatography-mass spectrometry according to the ISO 12966-4:2015 [50] method. The analysis of fatty acid methyl esters prepared according to ISO 12966-2:2017 [51] were performed on an Agilent Technologies (Palo Alto, CA, USA) gas chromatograph model 7890B coupled with a 5977A mass selective detector using a DB-23 Agilent Technologies capillary column (60 m × 0.25 mm i.d., film thickness 0.25 μm). The initial GC oven temperature was set at 50 °C for 1 min, increased to 200 °C at a rate of 25 °C/min, and further raised to 230 °C at a rate of 3 °C/min and finally kept at 230 °C for 7 min. The injector was maintained at 250 °C; injection volume was 1.0 μl and split ratio 1:50. Helium was used as carrier gas at a constant flow rate of 1.0 ml/min. The mass spectrometer was operated in the electron ionization mode (70 eV). Data acquisition was carried out in the scan mode (mass range: 50–400), solvent delay time was 5 min. The fatty acids peaks were identified by comparison of their retention times with retention times of known standards and by the mass spectra databases (Wiley 10th & NIST 2011 MS Library). A Supelco (Bellefonte, PA, USA) standard solution containing a mixture of 37 FAMEs was used for identification of peaks. The content of each fatty acid was expressed as percentage of total fatty acids.

### 2.3 Bioactive components investigation

The total tocopherol content (TTC) was determined by high-performance liquid chromatography (HPLC) according to the ISO 9936:2016 [52] method. Samples were analyzed using an HPLC (Sykam, Germany) equipped with fluorescence detector (FLD) and Nucleosil 100-5 NH2 (5 μm) column. The HPLC analyses were performed under isocratic conditions using a mobile phase composed of *n*-hexane and ethyl acetate (70:30, v/v). The FLD was set as follows: (λex. = 280 nm, λem. = 340 nm). Data were evaluated using clarity chromatography software (Data Apex).

Total phenols content was determinate spectrophotometrically using Folin-Ciocalteu's reagent according method described by Haiyan et al. [53] Spectrophotometric analysis was performed at 765 nm and results were expressed in milligrams of gallic acid per kilogram of oil.

Total carotenoids content (TCC) was estimated by measuring the absorbance of oil solution in cyclohexane at 445 nm according to British Standard method [54]. Carotenoid content was expressed as β-carotene in mg per kg of oil.

Total chlorophyll content, expressed as pheophytin a, was determined by measuring the oil absorbance at a wavelength of 667 nm by the method described by Pokorny et al. [55].

Total phytosterol content, expressed as β-sitosterol, was estimated by measuring the absorbance of oil solution in chloroform at 625 nm using Liebermann-Burchard's reagent according to method described by Araújo et al. [56].

### 2.4 Oxidative stability investigation

The oxidative stability of cold pressed sunflower oils was tested under accelerated storage conditions according to the methodology described by Gomes et al. [57], Maszewska et al. [58] and Naderi et al. [59]. The aliquot of 50 ml of each sample was placed in two glass vessels (internal diameter 88 mm, height 18 mm) stored in an oven at moderate temperatures (63 ± 2 °C) in the dark, in the manner that allowed free circulation of the heated air. The assessment of lipid oxidation progress was conducted after 0, 4 and 8 days. The oxidation products content was determined by the peroxide value (PV), anisidine value (AnV), conjugated dienes (CD) and conjugated trienes (CT) content. ISO official methods were used to determine PV [60], AnV [61] and conjugated dienes and trienes [62] of oil samples. The total oxidation index (totox) was calculated using the following equation (1) [63,64]:

(1) TOTOX=2PV+AnV

Also, the acid value (AV) was determined in the initial oil samples according to ISO 660:2009 [65].

All spectrophotometric measurements were made at a UV/VIS spectrophotometer model T80+ (PG Instruments Limited, London). Used chemicals and reagents were of analytical reagent grade. All analyses were performed in triplicate and results were presented as a mean value ± standard deviation. One way analysis of variance (ANOVA) with a post hoc Tukey's HSD test was used to determine significant differences among the data at the significance level p < 0.05.

### 2.5 Artificial neural network modeling

In this article, a Multilayer Perceptron Model (MLP) that consists of three layers (input, hidden and output) is evaluated. This architecture is used in prediction, and have been proven quite capable of approximating nonlinear functions [66], giving the reason for choosing it in this study.

The experimental database for ANN was randomly divided into: training, cross-validation and testing set of data (with 60%, 20% and 20%, respectively). It is advisable to use just one hidden layer, because the use of more layers exacerbates the problem of local minima [67,68]. The network has been trained with Levenberg-Marquardt algorithm due to its high accuracy in similar function approximation [68]. In order to find an optimal architecture, different number of neurons in the hidden layer was considered [66] and sum of squares error (SOS) for the network was calculated, as StatSoft Statistica's default [69]. The optimum number of hidden neurons was chosen upon minimizing the difference between predicted ANN values and desired outputs. The neural network calculation could be presented with a following equation (2) [70]:

(2) $$Y=f_{1}\left(W_{2}∙f_{2}\left(W_{1}∙X+B_{1}\right)+B_{2}\right)$$

where, coefficients associated with the hidden and the output layers (weights and biases) are grouped in matrices W_1_ and B_1_, and W_2_ and B_2_, respectively, Y is the matrix of the output variables, f_1_ and f_2_ are transfer functions in the hidden and output layers, respectively, and X is the matrix of input variables.

Weights and biases are determined during the training step which updates them using optimization procedures to minimize the error function between network and experimental outputs, evaluated according to the sum of squares, according to BFGS algorithm, which was used to speed up and stabilize convergence [66].

Training, testing and system implementation.

After defining the architecture of ANN, the training step is initiated. The training process was repeated several times in order to get the best performance of the ANN, due to a high degree of parameters variability. It was accepted that the successful training was achieved when learning and cross-validation curves (SOS vs. training cycles) approached zero.

#### 2.5.1 Global sensitivity analysis

Yoon's interpretation method was used to determine the relative influence of input variables [71]. This method was applied on the basis of the weight coefficients of the developed ANN.

#### 2.5.2 The accuracy of the ANN model

The numerical verification of the developed models was tested using coefficient of determination (*r*^2^), reduced chi-square (*χ*^2^), mean bias error (*MBE*), root mean square error (*RMSE*) and mean percentage error (*MPE*). These commonly used parameters can be calculated as follows equations (3), (4), (5), (6) [72]:

(3) $$\chi^{2}=\frac{\sum_{i=1}^{N}{\left(x_{\exp,i}-x_{pre,i}\right)}^{2}}{N-n}$$

(4) $$RMSE={\left[\frac{1}{N}∙\sum_{i=1}^{N}{\left(x_{pre,i}-x_{\exp,i}\right)}^{2}\right]}^{1/2}$$

(5) $$MBE=\frac{1}{N}∙\sum_{i=1}^{N}\left(x_{pre,i}-x_{\exp,i}\right)\text{,}$$

(6) $$MPE=\frac{100}{N}∙\sum_{i=1}^{N}\left(\frac{\left|x_{pre,i}-x_{\exp,i}\right|}{x_{\exp,i}}\right)$$

where *x*_exp,*i*_ stands for the experimental values and *x*_*pre*,*i*_ are the predicted values calculated by the model for these measurements. *N* and *n* are the number of observations and constants, respectively.

## 3 Results and discussion

### 3.1 Fatty acid composition

The fatty acid composition of cold pressed oils of sunflower hybrids grown in Serbia and Argentina is summarized in Table 1. High levels of unsaturated fatty acids, composed mainly of linoleic (C18:2) and oleic acid (C18:1) which contributed about 87% fatty acid composition was noticed. The mean content of linoleic acid in oil samples obtained in hybrids grown in Serbia and Argentina was 57.14 and 61.98%, while the oleic acid average content was 29.88% and 26.19%, respectively. Statistically significant difference (p < 0.05) was found between average contents of two most abundant fatty acids in oil samples grown in Serbia and Argentina. Among the other unsaturated fatty acid, palmitoleic (C16:1) and gadoleic acid (C20:1) were found in minor percentages (<0.2%) in some oil samples. Concerning the saturated fatty acids, palmitic acid was dominant with the content of approximately 6–7%, followed by stearic acid with 4%. Arachidic, behenic and lignoceric acid, were detected in all the samples in the small amounts, while myristic acid was appeared in 10 examined oils. The fatty acid composition was in accordance with the Codex Alimentarius Standards [73] for sunflower oils.

**Table 1:** Fatty acid composition of the investigated cold pressed oils.

Hybrid	Fatty acid (% m/m)									
	C14:0	C16:0	C16:1	C18:0	C18:1	C18:2	C20:0	C20:1	C22:0	C24:0
S1	nd	7.18 ± 0.12^klm^	nd	4.43 ± 0.05^jk^	30.43 ± 0.08^kl^	56.11 ± 0.32^cd^	0.32 ± 0.01^mno^	nd	1.01 ± 0.04^hijkl^	0.52 ± 0.02^hi^
S2	0.05 ± 0.00^c^	6.26 ± 0.05^de^	0.05 ± 0.00^bc^	3.68 ± 0.00^e^	31.07 ± 0.01^lm^	57.29 ± 0.03^ef^	0.23 ± 0.00^defg^	0.14 ± 0.00^cde^	0.85 ± 0.03^bcdef^	0.38 ± 0.01^abcd^
S3	nd	7.55 ± 0.10^no^	nd	4.04 ± 0.07^fg^	29.82 ± 0.21^k^	56.65 ± 0.19^de^	0.29 ± 0.01^ijklm^	nd	1.18 ± 0.11^mn^	0.47 ± 0.07^defghi^
S4	nd	6.52 ± 0.11^efgh^	nd	3.94 ± 0.08^f^	31.09 ± 0.10^lm^	56.75 ± 0.25^def^	0.24 ± 0.01^efgh^	0.18 ± 0.00^fg^	0.89 ± 0.01^defgh^	0.39 ± 0.04^abcde^
S5	nd	6.79 ± 0.12^hij^	nd	4.02 ± 0.06^f^	32.46 ± 0.08^op^	55.24 ± 0.26^bc^	0.26 ± 0.01^fghij^	nd	0.90 ± 0.01^defghi^	0.33 ± 0.00^ab^
S6	0.06 ± 0.00^d^	8.08 ± 0.13^p^	0.08 ± 0.00^e^	4.99 ± 0.11^l^	32.93 ± 0.04^p^	51.67 ± 0.20^a^	0.36 ± 0.01^pr^	0.16 ± 0.00^e^	1.20 ± 0.05^mn^	0.46 ± 0.05^defghi^
S7	nd	7.55 ± 0.12^no^	0.07 ± 0.00^de^	4.09 ± 0.05^fgh^	31.95 ± 0.13^no^	54.25 ± 0.24^b^	0.30 ± 0.01^jklmn^	0.18 ± 0.01^fg^	1.09 ± 0.02^jklm^	0.51 ± 0.05^ghi^
S8	nd	7.25 ± 0.17^lmn^	nd	3.05 ± 0.09^b^	27.83 ± 0.29^fgh^	60.31 ± 0.67^jk^	0.20 ± 0.01^bcd^	nd	0.87 ± 0.07^defg^	0.49 ± 0.05^efghi^
S9	0.06 ± 0.00^d^	7.55 ± 0.13^no^	0.07 ± 0.01^de^	3.37 ± 0.03^cd^	26.11 ± 0.24^e^	61.18 ± 0.36^k^	0.24 ± 0.00^defgh^	0.14 ± 0.00^bcde^	0.89 ± 0.00^defghi^	0.40 ± 0.04^bcdef^
S10	nd	6.65 ± 0.01^fghi^	0.08 ± 0.01^e^	2.46 ± 0.01^a^	28.19 ± 0.01^hi^	61.31 ± 0.06^k^	0.15 ± 0.01_a_	0.13 ± 0.01^abcd^	0.66 ± 0.03^a^	0.39 ± 0.03^abcd^
S11	nd	7.70 ± 0.01^o^	nd	4.93 ± 0.09^l^	28.02 ± 0.36^ghi^	57.66 ± 0.36^fg^	0.32 ± 0.01^lmno^	nd	0.95 ± 0.05^efghij^	0.42 ± 0.03^bcdefgh^
S12	nd	6.99 ± 0.38^jkl^	nd	3.02 ± 0.16^b^	28.62 ± 0.27^ij^	59.99 ± 0.40^j^	0.17 ± 0.02^ab^	0.11 ± 0.01^ab^	0.72 ± 0.05^abc^	0.37 ± 0.05^abcd^
S13	nd	8.08 ± 0.06^p^	nd	5.70 ± 0.01^m^	27.29 ± 0.03^f^	56.90 ± 0.14^def^	0.39 ± 0.01^rs^	nd	1.24 ± 0.09^n^	0.39 ± 0.04^abcde^
S14	nd	6.90 ± 0.09^ijk^	0.08 ± 0.01^e^	3.13 ± 0.06^b^	30.94 ± 0.22^lm^	57.29 ± 0.31^ef^	0.21 ± 0.00^cde^	0.14 ± 0.01^cde^	0.96 ± 0.05^efghij^	0.35 ± 0.04^abc^
S15	nd	7.47 ± 0.11^mno^	nd	4.44 ± 0.08^jk^	31.45 ± 0.18^mn^	54.51 ± 0.34^b^	0.34 ± 0.03^nop^	0.14 ± 0.01^bcde^	1.19 ± 0.04^mn^	0.46 ± 0.02^defghi^
A1	0.04 ± 0.00^b^	6.49 ± 0.01^efgh^	0.05 ± 0.00^bc^	4.40 ± 0.00^ijk^	27.40 ± 0.03^fg^	59.64 ± 0.02^ij^	0.33 ± 0.01^nop^	0.13 ± 0.01^abcd^	1.08 ± 0.08^jklm^	0.44 ± 0.01^cdefgh^
A2	nd	4.82 ± 0.01^a^	0.06 ± 0.00^cd^	3.33 ± 0.02^cd^	31.05 ± 0.07^lm^	58.57 ± 0.17^gh^	0.28 ± 0.01^ijkl^	0.20 ± 0.00^g^	1.14 ± 0.03^lmn^	0.55 ± 0.02^i^
A3	0.05 ± 0.00^c^	6.63 ± 0.04^fghi^	0.08 ± 0.00^e^	3.20 ± 0.03^bc^	23.27 ± 0.14^a^	65.12 ± 0.18^m^	0.23 ± 0.00^cdef^	0.11 ± 0.01^ab^	0.92 ± 0.01^defghi^	0.39 ± 0.01^abcde^
A4	nd	5.44 ± 0.14^b^	nd	4.09 ± 0.09^fg^	27.75 ± 0.73^fgh^	61.02 ± 0.93^k^	0.27 ± 0.01^ghijk^	0.14 ± 0.02^cde^	0.94 ± 0.05^defghi^	0.35 ± 0.01^abc^
A5	0.04 ± 0.00^b^	5.61 ± 0.01^b^	0.04 ± 0.00^b^	4.04 ± 0.04^fg^	25.42 ± 0.08^d^	63.26 ± 0.15^l^	0.27 ± 0.02^hijk^	0.14 ± 0.00^cde^	0.80 ± 0.01^abcd^	0.37 ± 0.01^abcd^
A6	0.04 ± 0.00^b^	6.40 ± 0.00^efg^	0.04 ± 0.00^b^	4.48 ± 0.02^k^	27.44 ± 0.02^fg^	59.65 ± 0.01^ij^	0.34 ± 0.01^op^	0.12 ± 0.01^abc^	1.03 ± 0.01^ijkl^	0.45 ± 0.04^cdefghi^
A7	0.03 ± 0.00^a^	5.76 ± 0.03^bc^	0.03 ± 0.00^a^	4.28 ± 0.02^hij^	29.00 ± 0.07^j^	58.94 ± 0.14^hi^	0.32 ± 0.02^lmno^	0.12 ± 0.00^abc^	1.01 ± 0.01^hijkl^	0.51 ± 0.02^fghi^
A8	0.03 ± 0.00a	5.59 ± 0.02^b^	nd	4.11 ± 0.03^gh^	27.32 ± 0.04^f^	61.31 ± 0.08^k^	0.27 ± 0.01^ghijk^	0.13 ± 0.01^abcd^	0.82 ± 0.01^bcde^	0.41 ± 0.00^bcdefg^
A9	0.04 ± 0.00b	6.01 ± 0.10^cd^	0.05 ± 0.00^bc^	3.12 ± 0.06^b^	24.36 ± 0.48^bc^	64.81 ± 0.66^m^	0.23 ± 0.01^cdef^	0.16 ± 0.00^ef^	0.84 ± 0.03^bcdef^	0.37 ± 0.02^abcd^
A10	nd	6.59 ± 0.03^efghi^	nd	3.35 ± 0.01^cd^	24.02 ± 0.05^bc^	64.41 ± 0.11^m^	0.19 ± 0.00^bc^	0.15 ± 0.01^de^	0.67 ± 0.02^a^	0.29 ± 0.01^a^
A11	nd	6.34 ± 0.06^def^	nd	4.88 ± 0.05^l^	23.80 ± 0.12^ab^	63.14 ± 0.21^l^	0.33 ± 0.00^mnop^	0.13 ± 0.01^abcd^	0.97 ± 0.00^fghij^	0.42 ± 0.03^bcdefgh^
A12	nd	5.52 ± 0.03^b^	nd	3.48 ± 0.01^d^	26.32 ± 0.05^e^	63.28 ± 0.12^l^	0.22 ± 0.01^cde^	0.14 ± 0.00^cde^	0.71 ± 0.02^ab^	0.33 ± 0.01^ab^
A13	nd	6.71 ± 0.02^ghij^	nd	5.62 ± 0.02^m^	24.65 ± 0.02^c^	61.08 ± 0.13^k^	0.41 ± 0.01^s^	nd	1.13 ± 0.07^klmn^	0.41 ± 0.00^bcdefg^
A14	nd	5.68 ± 0.02^bc^	nd	3.67 ± 0.01^e^	24.61 ± 0.03^c^	64.42 ± 0.08^m^	0.24 ± 0.00^efgh^	0.11 ± 0.01^ab^	0.86 ± 0.06^cdefg^	0.40 ± 0.03^bcde^
A15	nd	6.34 ± 0.01^def^	nd	4.22 ± 0.02^hi^	26.43 ± 0.07^e^	61.13 ± 0.02^k^	0.31 ± 0.01^klmno^	0.14 ± 0.01^cde^	0.99 ± 0.00^ghijk^	0.43 ± 0.04^bcdefgh^
Serbia	0.06 ± 0.01^a^	7.23 ± 0.55^a^	0.07 ± 0.01^a^	3.95 ± 0.87^a^	29.88 ± 2.07^a^	57.14 ± 2.70^a^	0.27 ± 0.07^a^	0.15 ± 0.02^a^	0.97 ± 0.18^a^	0.42 ± 0.06^a^
Argentina	0.04 ± 0.01^b^	6.00 ± 0.55^b^	0.05 ± 0.01^b^	4.02 ± 0.69^a^	26.19 ± 2.17^b^	61.98 ± 2.22^b^	0.28 ± 0.06^a^	0.14 ± 0.00^a^	0.93 ± 0.14^a^	0.41 ± 0.07^a^

nd - not detected.

Different lower-case letters in the same column indicate significantly different values (p < 0.05), according to post hoc Tukey's HSD.

### 3.2 Bioactive components investigation

The presence of bioactive compounds in cold pressed oils is desirable concerning their beneficial effect on the oil's oxidative properties. Table 2 shows bioactive compounds content in the investigated oil samples. Compared to other bioactive compounds present in sunflower oil, total sterols content were found in the highest concentration (on average 1544.36 ± 162.24 mg/kg), followed by the total tocopherols content (518.24 ± 96.06 mg/kg). Approximate average total phenols and carotenoids concentrations were noticed in the examined samples, 9.43 ± 0.83 mg/kg and 7.54 ± 2.30 mg/kg, respectively. Low average total chlorophyll content was determined in the samples, only 0.99 ± 0.14 mg/kg. The obtained values are in accordance with previous studies [37,40].

**Table 2:** Total phenols content, total tocopherol content, total carotenoids content, total chlorophyll content and total phytosterols content of the investigated cold pressed oils.

Hybrid	Total phenols content (mg/kg)	Tocopherols content (mg/kg)			Total carotenoids content (as β-carotene) (mg/kg)	Total chlorophyll content (as pheophytin a) (mg/kg)	Total phytosterols content (as β-sitosterol) (mg/kg)
		α-tocopherol(mg/kg)	β-tocopherol(mg/kg)	Total tocopherols content (mg/kg)			
S1	8.88 ± 0.08^abcd^	447.26 ± 11.65^cdef^	<0.59^a^	447.26 ± 11.65^cde^	4.30 ± 0.04^a^	0.58 ± 0.02^abc^	1370.00 ± 45.83^bcd^
S2	7.88 ± 0.12^a^	674.61 ± 7.10^pr^	<0.59^a^	674.61 ± 7.10^mn^	5.84 ± 0.01^abcd^	0.80 ± 0.02^bcdef^	1356.67 ± 45.09^abcd^
S3	9.30 ± 0.14^abcde^	469.91 ± 4.04^efg^	<0.59^a^	469.91 ± 4.04^efg^	6.89 ± 0.08^abcdefgh^	0.77 ± 0.03^bcde^	1536.67 ± 37.86^ghij^
S4	9.56 ± 0.22^abcde^	719.41 ± 5.16^r^	<0.59^a^	719.41 ± 5.16^n^	7.74 ± 0.08^bcdefghi^	1.02 ± 0.03^cdefgh^	1730.00 ± 55.68^mno^
S5	9.80 ± 0.24^abcde^	684.80 ± 8.62^pr^	<0.59^a^	684.80 ± 8.62^mn^	4.96 ± 0.01^ab^	1.08 ± 0.06^cdefgh^	1656.67 ± 30.55^klmn^
S6	8.77 ± 0.45^abcd^	466.41 ± 8.78^efg^	<0.59^a^	466.41 ± 8.78^efg^	6.46 ± 0.07^abcdef^	0.71 ± 0.04^abcde^	1550.67 ± 45.62^hijk^
S7	9.73 ± 0.51^abcde^	539.65 ± 21.14^jklm^	<0.59^a^	539.65 ± 21.14^hij^	8.02 ± 0.07^cdefghi^	0.91 ± 0.02^cdefg^	1433.33 ± 30.55^cdefg^
S8	9.38 ± 0.41^abcde^	534.72 ± 13.40^jkl^	<0.59^a^	534.72 ± 13.40^hi^	6.45 ± 0.07^abcdef^	1.22 ± 0.02^efgh^	1650.00 ± 36.06^klmn^
S9	8.02 ± 0.38^ab^	544.37 ± 8.30^klm^	<0.59^a^	544.37 ± 8.30^hij^	7.05 ± 0.17^abcdefgh^	0.17 ± 0.01^a^	1720.00 ± 20.00^mno^
S10	9.20 ± 0.67^abcd^	564.65 ± 10.46^klm^	<0.59^a^	564.65 ± 10.46^ijk^	9.52 ± 0.20^ghi^	0.75 ± 0.00^bcde^	1346.67 ± 37.86^abc^
S11	8.16 ± 0.47^abc^	456.53 ± 7.06^defg^	<0.59^a^	456.53 ± 7.06^de^	10.53 ± 0.19^ijk^	0.64 ± 0.00^abcd^	1253.33 ± 30.55^a^
S12	9.26 ± 0.78^abcde^	471.67 ± 6.56^efgh^	<0.59^a^	471.67 ± 6.56^efg^	9.41 ± 0.08^fghi^	0.60 ± 0.01^abcd^	1463.33 ± 15.28^defgh^
S13	9.86 ± 0.82^abcde^	403.60 ± 2.84^abc^	<0.59^a^	403.60 ± 2.84^abc^	6.80 ± 0.07^abcdefg^	1.15 ± 0.02^defgh^	1663.33 ± 15.28^lmn^
S14	7.74 ± 0.32^a^	430.50 ± 8.92^bcde^	<0.59^a^	430.50 ± 8.92^bcde^	9.85 ± 0.13^hij^	0.31 ± 0.02^ab^	1503.33 ± 15.28^ghi^
S15	8.91 ± 0.30^abcd^	458.68 ± 10.54^efg^	<0.59^a^	458.68 ± 10.54^def^	9.10 ± 0.13^efghi^	0.29 ± 0.01^ab^	1353.33 ± 35.12^abcd^
A1	9.51 ± 0.78^abcde^	395.95 ± 17.64^ab^	15.48 ± 4.74^bc^	411.43 ± 19.81^abcd^	5.09 ± 0.20^abc^	0.91 ± 0.31^cdefg^	1453.33 ± 35.12^cdefgh^
A2	9.22 ± 0.84^abcd^	374.27 ± 17.60^a^	16.99 ± 2.75^bcde^	391.26 ± 17.56^ab^	8.60 ± 0.30^defghi^	1.28 ± 0.17^efgh^	1483.33 ± 30.55^efghi^
A3	8.91 ± 0.62^abcd^	373.04 ± 8.51^a^	<0.59^a^	373.04 ± 8.51^a^	6.64 ± 0.53^abcdefg^	1.08 ± 0.03^cdefgh^	1620.00 ± 36.06^jklm^
A4	10.31 ± 1.01^bcde^	639.04 ± 13.41^op^	25.28 ± 1.99^g^	664.32 ± 14.99^m^	10.03 ± 0.54^ij^	1.37 ± 0.35^ghi^	1786.67 ± 25.17^op^
A5	11.60 ± 1.31^e^	585.05 ± 16.48^mn^	15.52 ± 2.18^bcd^	600.57 ± 15.82^kl^	6.15 ± 2.46^abcde^	1.05 ± 0.03^cdefgh^	1723.33 ± 49.33^mno^
A6	10.34 ± 0.89^bcde^	410.33 ± 17.51^abcd^	12.81 ± 3.64^bc^	423.13 ± 20.95^bcde^	12.80 ± 1.12^jk^	1.15 ± 0.16^defgh^	1660.00 ± 43.59^klmn^
A7	9.58 ± 0.78^abcde^	370.67 ± 0.86^a^	12.46 ± 2.15^bc^	383.13 ± 1.75^ab^	8.63 ± 3.34^defghi^	1.22 ± 0.48^efgh^	1496.67 ± 32.15^fghi^
A8	9.26 ± 0.75^abcde^	623.43 ± 11.25^no^	24.32 ± 1.76^fg^	647.75 ± 13.00^lm^	6.78 ± 1.48^abcdefg^	0.52 ± 0.21^abc^	1760.00 ± 36.06^no^
A9	10.08 ± 1.12^abcde^	493.93 ± 13.33^fghij^	18.87 ± 1.70^cdefg^	512.80 ± 14.02^gh^	13.08 ± 0.22^k^	1.43 ± 0.09^ghi^	1883.33 ± 25.17^p^
A10	10.61 ± 0.87^de^	530.38 ± 11.32^ijkl^	11.76 ± 1.52^b^	542.15 ± 12.51^hij^	9.10 ± 1.60^efghi^	1.52 ± 0.20^hi^	1376.67 ± 25.17^bcde^
A11	9.91 ± 0.75^abcde^	518.53 ± 1.44^hijk^	23.26 ± 0.27^efg^	541.79 ± 1.55^hij^	4.23 ± 0.37^a^	1.36 ± 0.10^fghi^	1316.67 ± 37.86^ab^
A12	9.54 ± 0.85^abcde^	563.08 ± 3.58^klm^	17.49 ± 1.89^bcde^	580.57 ± 5.03^ijk^	4.86 ± 0.40^ab^	1.90 ± 0.27^i^	1523.33 ± 32.15^ghij^
A13	10.42 ± 1.34^cde^	484.59 ± 43.25^fghi^	21.96 ± 1.56^defg^	506.55 ± 44.16^efg^	5.51 ± 0.36^abc^	1.43 ± 0.14^ghi^	1690.00 ± 26.46^lmno^
A14	9.39 ± 0.86^abcde^	572.00 ± 22.61^lm^	16.62 ± 4.49^bcd^	588.62 ± 19.40^jk^	5.96 ± 0.87^abcd^	1.27 ± 0.45^efgh^	1583.33 ± 30.55^ijkl^
A15	9.67 ± 0.87^abcde^	494.70 ± 27.38^ghij^	18.69 ± 5.46^cdef^	513.39 ± 32.16^fg^	5.93 ± 0.45^abcd^	1.23 ± 0.16^efgh^	1386.67 ± 32.15^bcdef^
Serbia	8.96 ± 0.71^a^	524.45 ± 98.59^b^	<0.59^a^	524.45 ± 98.59^a^	7.53 ± 1.85^a^	0.73 ± 0.31^a^	1505.82 ± 152.96^a^
Argentina	9.89 ± 0.69^b^	495.27 ± 92.47^a^	16.77 ± 6.27^b^	512.03 ± 96.50^a^	7.56 ± 2.75^a^	1.25 ± 0.31^b^	1582.89 ± 167.17^a^

Values are means ± standard deviation (n = 3).

Different lower-case letters in the same column indicate significantly different values (p < 0.05), according to post hoc Tukey's HSD.

Limit of detection for β-tocopherol was 0.59 mg/kg.

#### 3.2.1 Total tocopherols and phenols content

Tocopherols affect the biological value of the oil and contribute to its oxidative stability. In the average values of the TTC no significant (p < 0.05) difference was found, namely samples from Serbia had on average only 2.42% higher TTC compared to the Argentina samples. However, a fairly large range has been observed, TTC content found in the samples grown in Serbia ranged from 403.60 ± 2.84 to 719.41 ± 5.16 mg/kg, while in the case of Argentina samples TTC content amounted from 373.04 ± 8.51 to 664.32 ± 14.99 mg/kg. Similar values were reported by Dimić et al. [40] and Gliszczyńska-Świgło et al. [74], where tocopherols content in cold pressed sunflower oil were ranged from 485.23 ± 0.26 to 589.50 ± 0.71 mg/kg and 535 ± 8 mg/kg, respectively. Obtained results also were in accordance with Codex-Apartment 210–1999 [73] for sunflower oil.

Phenolic compounds have a strong antioxidative effect [75,76]. Compared to other oils, sunflower oil contains insignificant amounts of phenolic compounds, e.g., the content of phenolic compounds in olive oil can reach as much as 1000 mg/kg [77], while sunflower oil commonly has about 10 mg/kg [26]. However, low concentrations of phenolic compounds also affect the stability of the oil [30,31]. In the tested samples, the content of phenolic compounds ranged from 7.74 ± 0.32 mg/kg found in S14 sample to 11.60 ± 1.31 mg/kg, observed in A5 sample (Table 2). The obtained results were in line with previous research [40]. Concerning the average content of total phenolic compounds, a statistically significant difference (p˂0.05) was found among the samples grown on the territory of Serbia and Argentina.

#### 3.2.2 Total carotenoids and chlorophylls content

Carotenoids, yellow pigments, and chlorophylls, green pigments, in addition to affecting the sensory quality of unrefined oils and color formation [78,79], also affect other aspects of quality, such as oil stability and shelf life [80,81]. Carotenoids present in unrefined oils exhibit antioxidant activity, similar to tocopherols [1,36,82]. Some research also confirmed the antioxidant activity of chlorophylls [83]. Total carotenoids content in examined oil samples ranged from 4.23 ± 0.37 to 13.08 ± 0.22 mg/kg (Table 2). The average total carotenoids content of samples grown in Serbia (7.53 ± 1.85 mg/kg) was not statistically different (p˂0.05) compared to samples from Argentina (7.56 ± 2.75 mg/kg). Total chlorophylls content in the examined oil samples was found in significantly lower amounts, on average 0.99 ± 0.40 mg/kg (Table 2), characteristic for unrefined sunflower oil. Tuberoso et al. [37] reported total chlorophylls content in sunflower oil of 2.3 ± 0.1 mg/kg, while Dimić et al. [40] found lower chlorophylls content (from 0.00 ± 0.00 to 1.21 ± 0.01 mg/kg). The average total chlorophyll content of samples grown in Serbia (0.73 ± 0.31 mg/kg) was statistically different (p˂0.05) in comparison to samples from Argentina (1.25 ± 0.31 mg/kg).

#### 3.2.3 Total phytosterols content

The phytosterols have a significant role in oxidative stability of oil, since sterols can delay oil degradation when subjected to prolong heating [84]. No significant difference was found in the total sterols content of samples grown in Serbia and Argentina, and the average values were 1505.82 ± 152.96 mg/kg (Serbia) and 1582.89 ± 167.17 mg/kg (Argentina). The obtained values are in accordance with the results reported by Ayerdi-Gotor et al. [85]. Namely, they determined the total sterols content in the range of 1250–7650 mg/kg in a wide group of sunflower hybrids and inbred lines. On the other hand, Piironen et al. [86] reported higher total phytosterols content in crude sunflower oil, ranging from 3740 to 7250 mg/kg. Significantly higher values, compared to results obtained in this paper, may be the result of different investigation methods.

### 3.3 Oxidative stability

Acid value indicates the degree of hydrolytic changes of the oil, i. e., free acidity. All examined oil samples had acid values less than 1 mg KOH/g oil (Table 3), indicating good oil quality, significantly better compared to results obtained by Konuskan et al. [87] (1.62 mg KOH/g) or Codex Alimentarius [73] recommendations for cold pressed sunflower oil (4.00 mg KOH/g). No significant difference in average AV values was noticed between samples grown in Serbia and Argentina.

**Table 3:** Acid value (AV), conjugated dienes (CD) and conjugated trienes (CT) content of the investigated cold pressed oils during accelerated stability test.

Hybrid	AV	CD			CT		
	(mgKOH/g)	0^th^ day	4th day	8th day	0^th^ day	4th day	8th day
S1	0.67 ± 0.04^cdefg^	2.61 ± 0.03^cdefA^	5.33 ± 0.45^dA^	14.62 ± 2.58^defB^	0.11 ± 0.01^abA^	0.35 ± 0.02^efghijB^	0.52 ± 0.01^abcdeC^
S2	0.72 ± 0.07^cdefgh^	2.51 ± 0.04^bcdA^	7.02 ± 0.97^efA^	16.59 ± 4.24^fgB^	0.22 ± 0.01^defgA^	0.44 ± 0.01^mnB^	0.62 ± 0.04^cdefghijC^
S3	0.70 ± 0.06^cdefg^	2.30 ± 0.01^aA^	4.88 ± 0.38^cdA^	19.98 ± 4.51^gB^	0.14 ± 0.01^bcA^	0.34 ± 0.02^efghiB^	0.50 ± 0.01^abcdC^
S4	0.41 ± 0.02^ab^	2.75 ± 0.02^fghA^	3.97 ± 0.18^abcB^	4.56 ± 0.24^aC^	0.18 ± 0.00^cdA^	0.30 ± 0.01^cdeB^	0.41 ± 0.02^aC^
S5	0.65 ± 0.05^cdefg^	2.71 ± 0.03^efgA^	3.71 ± 0.40^abB^	9.46 ± 0.49^abcC^	0.23 ± 0.01^efghA^	0.31 ± 0.01^defgB^	0.42 ± 0.01^abC^
S6	0.56 ± 0.07^abcd^	2.51 ± 0.01^bcdA^	4.85 ± 0.87^cdA^	16.33 ± 2.14^efgB^	0.15 ± 0.01^bcA^	0.34 ± 0.00^efghijB^	0.39 ± 0.01^aC^
S7	0.57 ± 0.04^abcd^	2.72 ± 0.06^efghA^	4.54 ± 0.32^bcdA^	16.71 ± 2.87^fgB^	0.15 ± 0.01^bcA^	0.39 ± 0.01^hijklmB^	0.49 ± 0.01^abcdC^
S8	0.71 ± 0.03^cdefg^	3.29 ± 0.01^kA^	4.81 ± 0.68^cdA^	10.12 ± 1.87^cdB^	0.25 ± 0.01^fghiA^	0.40 ± 0.02^ijklmB^	0.48 ± 0.00^abcC^
S9	0.81 ± 0.23^efghi^	3.10 ± 0.00^jA^	4.23 ± 0.21^abcA^	10.04 ± 1.47^cdB^	0.29 ± 0.01^ijA^	0.30 ± 0.01^cdeA^	0.58 ± 0.05^bcdefghB^
S10	0.67 ± 0.08^cdefg^	2.76 ± 0.01^fghA^	3.36 ± 0.14^aA^	9.89 ± 0.43^cdB^	0.22 ± 0.01^defgA^	0.38 ± 0.01^hijklmB^	0.78 ± 0.07^jklmC^
S11	0.60 ± 0.07^abcde^	2.63 ± 0.06^defA^	4.83 ± 0.25^cdB^	11.56 ± 1.32^cdeC^	0.27 ± 0.01^ghijA^	0.41 ± 0.02^jklmB^	0.95 ± 0.06^nC^
S12	0.68 ± 0.07^cdefg^	3.04 ± 0.01^jA^	4.04 ± 0.18^abcB^	8.37 ± 0.59^abcC^	0.31 ± 0.04^jkA^	0.38 ± 0.01^hijklmA^	0.84 ± 0.07^mnB^
S13	0.86 ± 0.09^fghi^	3.44 ± 0.04^kA^	4.55 ± 0.34^bcdA^	10.40 ± 1.40^cdB^	0.28 ± 0.01^hijA^	0.38 ± 0.00^ghijklmB^	0.68 ± 0.04^fghijklC^
S14	0.95 ± 0.11^i^	2.31 ± 0.05^aA^	4.79 ± 0.25^bcdB^	12.56 ± 1.52^cdefC^	0.11 ± 0.01^abA^	0.37 ± 0.01^fghijklB^	0.68 ± 0.07^efghijkC^
S15	0.60 ± 0.09^abcde^	2.40 ± 0.01^abA^	4.49 ± 0.46^bcdB^	8.46 ± 0.49^abcC^	0.24 ± 0.01^efghA^	0.31 ± 0.01^defgB^	0.60 ± 0.03^cdefghiC^
A1	0.88 ± 0.15^ghi^	3.31 ± 0.10^kA^	6.93 ± 0.15^efB^	10.84 ± 0.02^cdC^	0.18 ± 0.00^cdA^	0.20 ± 0.01^bA^	0.47 ± 0.01^abcB^
A2	0.94 ± 0.04^hi^	2.73 ± 0.01^efghA^	4.47 ± 0.00^bcdB^	8.96 ± 0.01^abcC^	0.08 ± 0.00^aA^	0.13 ± 0.01^aB^	0.46 ± 0.01^abcC^
A3	0.78 ± 0.01^defghi^	2.88 ± 0.08^hiA^	6.94 ± 0.05^efB^	10.83 ± 0.02^cdC^	0.19 ± 0.07^cdeA^	0.23 ± 0.01^bcA^	0.76 ± 0.09^ijklmB^
A4	0.50 ± 0.04^abc^	2.84 ± 0.02^ghiA^	8.60 ± 0.03^ghB^	10.27 ± 0.01^cdC^	0.22 ± 0.00^defgA^	0.39 ± 0.04^hijklmB^	0.72 ± 0.06^hijklmC^
A5	0.57 ± 0.02^abcd^	2.82 ± 0.02^ghiA^	5.00 ± 0.02^cdB^	9.68 ± 0.01^bcC^	0.27 ± 0.00^ghijA^	0.33 ± 0.05^efghA^	0.54 ± 0.01^abcdefgB^
A6	0.48 ± 0.02^abc^	3.06 ± 0.08^jA^	7.07 ± 0.01^efB^	9.49 ± 0.03^bcC^	0.23 ± 0.00^defgA^	0.45 ± 0.01^mnB^	0.54 ± 0.13^abcdefB^
A7	0.49 ± 0.08^abc^	2.54 ± 0.02^bcdA^	6.77 ± 0.02^efB^	4.76 ± 0.02^abC^	0.21 ± 0.01^defA^	0.25 ± 0.00^bcdB^	0.66 ± 0.01^efghijkC^
A8	0.40 ± 0.02^a^	2.58 ± 0.05^cdeA^	4.86 ± 0.08^cdB^	10.04 ± 0.01^cdC^	0.25 ± 0.01^fghiA^	0.32 ± 0.07^efghA^	0.65 ± 0.00^defghijkB^
A9	0.57 ± 0.01^abcd^	2.73 ± 0.15^efghA^	7.20 ± 0.02^efB^	10.54 ± 0.02^cdC^	0.24 ± 0.01^efghA^	0.43 ± 0.02^lmB^	0.78 ± 0.06^klmC^
A10	0.61 ± 0.02^abcde^	2.49 ± 0.03^bcdA^	8.29 ± 0.02^ghB^	10.37 ± 0.02^cdC^	0.28 ± 0.03^hijA^	0.50 ± 0.00^nB^	0.80 ± 0.09^klmnC^
A11	0.64 ± 0.06^bcdef^	2.85 ± 0.02^ghiA^	9.23 ± 0.01^hB^	9.71 ± 0.02^cdC^	0.31 ± 0.01^jA^	0.50 ± 0.00^nB^	0.84 ± 0.02^lmnC^
A12	0.69 ± 0.05^cdefg^	2.96 ± 0.05^ijA^	6.56 ± 0.00^efB^	9.87 ± 0.01^cdC^	0.36 ± 0.01^kA^	0.42 ± 0.05^klmA^	0.80 ± 0.02^klmnB^
A13	0.55 ± 0.02^abcd^	2.81 ± 0.03^ghiA^	7.64 ± 0.01^fgB^	9.08 ± 0.05^abcC^	0.31 ± 0.00^jA^	0.35 ± 0.01^efghijkA^	0.70 ± 0.10^ghijklmB^
A14	0.58 ± 0.01^abcde^	2.73 ± 0.05^efghA^	8.63 ± 0.02^ghB^	9.18 ± 0.02^abcC^	0.30 ± 0.01^ijA^	0.39 ± 0.01^hijklmB^	0.59 ± 0.02^cdefghC^
A15	0.53 ± 0.01^abc^	2.47 ± 0.02^bcA^	6.50 ± 0.00^eB^	8.76 ± 0.01^abcC^	0.27 ± 0.01^ghijA^	0.30 ± 0.00^defB^	0.58 ± 0.01^cdefghC^
Serbia	0.68 ± 0.13^a^	2.74 ± 0.34^a^	4.63 ± 0.84^a^	11.98 ± 4.10^a^	0.21 ± 0.07^a^	0.36 ± 0.04^a^	0.60 ± 0.16^a^
Argentina	0.62 ± 0.15^a^	2.79 ± 0.22^a^	6.98 ± 1.41^b^	9.49 ± 1.47^b^	0.25 ± 0.07^a^	0.35 ± 0.11^a^	0.66 ± 0.12^a^

Values are means ± standard deviation (n = 3).

Different lower-case letters in the same column indicate significantly different values (p < 0.05), according to post hoc Tukey's HSD.

Different upper-case letters in same raw indicate significantly different values of inidividual oxidative parameter (CD, CT) between days during Schaal oven test (p < 0.05), according to post hoc Tukey's HSD.

CD content in the initial samples ranged from 2.30 ± 0.01 (determined in the S3) to 3.44 ± 0.04 (S13). In S13 sample the highest peroxide value and totox index (Table 4) was found, 3.64 ± 0.02 mmol/kg and 8.00 ± 0.04, respectively. Statistically significant correlation between peroxide value and conjugated dienes content (R = 0.52; p = 0.03) and between totox index and conjugated dienes content (R = 0.56; p = 0.01) in the initial samples was found. Stronger CD-PV and CD-totox correlations were found during whole test (0^th^, 4th and 8th day) exposure and amounted R = 0.83 (p = 0.00) for both correlations. CD content indicates primary oxidation products, as well as PV [[88], [89], [90]] wherefore significant correlation was noticed. Totox index indicates overall oxidative changes, but a higher share is occupied by primary oxidation products (Eq. (1)), what was a main reason of the significant correlation.

**Table 4:** Peroxide value (PV), anisidine value (AnV) and total oxidation index (TOTOX) of the investigated cold pressed oils during accelerated stability test.

Hybrid	PV			AnV			TOTOX		
	0^th^ day	4th day	8th day	0^th^ day	4th day	8th day	0^th^ day	4th day	8th day
S1	2.96 ± 0.05^opA^	32.53 ± 0.08^hijkB^	72.39 ± 3.72^cdefghC^	1.13 ± 0.61^hijkA^	1.45 ± 0.12^defgA^	2.18 ± 0.42^bcdefghA^	7.05 ± 0.70^klmA^	66.51 ± 0.28^jklmB^	146.96 ± 7.65^cdefghiC^
S2	2.44 ± 0.08^jklmnA^	33.22 ± 0.64^jkB^	72.77 ± 3.50^cdefghC^	1.13 ± 0.10^hijkA^	1.26 ± 0.10^bcdefgA^	1.65 ± 0.07^abcdeB^	6.01 ± 0.12^hijA^	67.70 ± 1.38^klmnB^	147.19 ± 6.94^cdefghiC^
S3	2.11 ± 0.08^efghijA^	32.56 ± 0.54^hijkB^	71.90 ± 3.20^cdefghC^	1.05 ± 0.10^ghijA^	1.68 ± 0.22^efghB^	2.62 ± 0.08^efghiC^	5.27 ± 0.21^efghA^	66.80 ± 1.21^jklmB^	146.42 ± 6.48^cdefghiC^
S4	1.96 ± 0.05^cdefghA^	36.90 ± 0.09^mB^	86.99 ± 2.61^iC^	0.84 ± 0.08^fghiA^	1.02 ± 0.20^abcdeAB^	1.87 ± 0.57^abcdefB^	4.76 ± 0.17^defgA^	74.82 ± 0.02^oB^	175.85 ± 4.96^jC^
S5	2.16 ± 0.19^ghijkA^	30.95 ± 0.74^efghiB^	73.22 ± 4.20^cdefghC^	0.50 ± 0.05^cdefA^	0.98 ± 0.15^abcdeB^	3.60 ± 0.21^ijkC^	4.82 ± 0.35^defgA^	62.88 ± 1.33^fghijB^	150.04 ± 8.61^defghiC^
S6	2.99 ± 0.01^pA^	31.04 ± 0.65^efghiB^	70.29 ± 3.50^cdefC^	0.65 ± 0.07^efgA^	0.91 ± 0.16^abcdeA^	2.67 ± 0.25^efghiB^	6.64 ± 0.07^jklA^	62.99 ± 1.46^fghijB^	143.25 ± 7.25^cdefgC^
S7	2.43 ± 0.15^jklmnA^	31.45 ± 1.37^efghijB^	68.42 ± 3.10^cdefC^	1.36 ± 0.10^jklA^	1.72 ± 0.41^efghAB^	2.51 ± 0.40^defghiB^	6.22 ± 0.35^ijkA^	64.62 ± 2.33^hijklB^	139.35 ± 5.81^cdefgC^
S8	3.43 ± 0.01^rA^	31.96 ± 0.75^ghijB^	74.85 ± 4.50^defghC^	1.03 ± 0.20^ghijA^	1.76 ± 0.50^efghAB^	2.43 ± 0.30^defghiB^	7.89 ± 0.18^mnA^	65.68 ± 2.00^ijklB^	152.13 ± 9.30^efghiC^
S9	2.53 ± 0.05^lmnA^	34.45 ± 0.18^klB^	73.12 ± 3.20^cdefghC^	1.01 ± 0.09^ghijA^	1.38 ± 0.32^cdefgAB^	1.92 ± 0.25^abcdefB^	6.07 ± 0.07^hijA^	70.28 ± 0.57^mnB^	148.16 ± 6.65^cdefghiC^
S10	2.73 ± 0.11^nopA^	46.20 ± 1.20^pB^	80.77 ± 6.20^ghiC^	2.30 ± 0.12^mA^	2.52 ± 0.65^hAB^	3.60 ± 0.42^ijkB^	7.77 ± 0.30^mnA^	94.92 ± 1.75^rB^	165.14 ± 12.82^hijC^
S11	2.64 ± 0.21^mnopA^	41.40 ± 0.98^noB^	75.20 ± 5.23^efghC^	0.84 ± 0.12^fghA^	1.12 ± 0.12^abcdefA^	1.20 ± 0.36^abcA^	6.12 ± 0.54^hijA^	83.92 ± 2.08^pB^	151.60 ± 10.82^defghiC^
S12	2.97 ± 0.10^pA^	39.77 ± 1.01^nB^	81.36 ± 2.11^hiC^	1.27 ± 0.01^ijklA^	2.01 ± 0.41^fghB^	3.25 ± 0.03^hijC^	7.21 ± 0.21^lmnA^	81.55 ± 1.61^pB^	165.97 ± 4.19^ijC^
S13	3.64 ± 0.02^rA^	33.14 ± 0.85^jkB^	68.91 ± 2.08^cdefC^	0.71 ± 0.02^efghA^	2.13 ± 0.35^ghB^	4.61 ± 0.02^kC^	8.00 ± 0.04^nA^	68.41 ± 2.05^lmnB^	142.43 ± 4.18^cdefgC^
S14	2.27 ± 0.33^hijklA^	41.95 ± 1.23^oB^	77.37 ± 4.23^fghiC^	0.12 ± 0.04^abcA^	1.35 ± 0.12^cdefgB^	3.14 ± 0.39^ghijC^	4.66 ± 0.66^cdefA^	85.25 ± 2.58^pB^	157.88 ± 8.85^ghijC^
S15	1.91 ± 0.06^bcdefgA^	27.29 ± 0.75^cB^	67.38 ± 3.23^cdeC^	0.63 ± 0.07^defgA^	1.68 ± 0.45^efghB^	2.31 ± 0.42^cdefghB^	4.44 ± 0.16^bcdeA^	56.26 ± 1.95^cdeB^	137.07 ± 6.88^cdefC^
A1	2.48 ± 0.20^klmnA^	27.52 ± 0.64^cdB^	68.22 ± 1.80^cdefC^	3.01 ± 0.02^nA^	3.84 ± 0.68^iA^	4.02 ± 0.78^jkA^	7.98 ± 0.42^nA^	58.87 ± 1.72^defB^	140.46 ± 4.06^cdefgC^
A2	2.12 ± 0.05^fghijA^	15.10 ± 0.57^aB^	52.20 ± 1.22^aC^	0.34 ± 0.05^abcdeA^	0.85 ± 0.10^abcdeB^	1.35 ± 0.18^abcdC^	4.57 ± 0.13^cdefA^	31.05 ± 1.09^aB^	105.74 ± 2.62^aC^
A3	1.77 ± 0.01^abcdefA^	30.10 ± 0.06^efgB^	72.16 ± 0.29^cdefghC^	1.59 ± 0.04^lA^	4.32 ± 0.54^iB^	4.68 ± 0.74^kB^	5.13 ± 0.06^efgA^	64.52 ± 0.62^ghijklB^	149.00 ± 1.16^defghiC^
A4	1.82 ± 0.06^abcdefgA^	32.65 ± 0.01^ijkB^	73.18 ± 0.09^cdefghC^	0.46 ± 0.01^bcdefA^	1.25 ± 0.20^bcdefgB^	1.98 ± 0.41^abcdefgC^	4.11 ± 0.13^abcdA^	66.56 ± 0.18^jklmB^	148.34 ± 0.37^cdefghiC^
A5	2.01 ± 0.06^defghiA^	29.46 ± 0.28^deB^	71.33 ± 0.60^cdefgC^	1.51 ± 0.01^klA^	2.05 ± 0.42^fghAB^	2.87 ± 0.54^fghijB^	5.54 ± 0.12^ghiA^	60.98 ± 0.50^fghB^	145.53 ± 1.53^cdefghC^
A6	2.11 ± 0.05^efghijA^	26.96 ± 0.49^cB^	63.53 ± 0.38^bcC^	0.55 ± 0.03^defA^	0.86 ± 0.05^abcdeA^	1.75 ± 0.32^abcdefB^	4.78 ± 0.12^defgA^	54.78 ± 0.96^cdB^	128.81 ± 0.97^bcC^
A7	1.59 ± 0.02^abA^	20.72 ± 0.09^bB^	54.15 ± 1.55^abC^	0.21 ± 0.05^abcdA^	0.45 ± 0.09^abcA^	1.54 ± 0.41^abcdeB^	3.39 ± 0.09^aA^	41.89 ± 0.17^bB^	109.85 ± 2.92^abC^
A8	1.54 ± 0.03^aA^	25.78 ± 0.12^cB^	77.48 ± 3.88^fghiC^	0.21 ± 0.09^abcdA^	0.65 ± 0.10^abcdB^	1.67 ± 0.25^abcdeC^	3.29 ± 0.10^aA^	52.21 ± 0.34^cB^	156.63 ± 7.88^fghijC^
A9	2.28 ± 0.10^hijklA^	35.64 ± 0.40^lmB^	70.60 ± 4.01^cdefC^	0.07 ± 0.02^abA^	0.23 ± 0.03^aA^	1.02 ± 0.14^abB^	4.63 ± 0.18^cdefA^	71.51 ± 0.78^noB^	142.21 ± 8.10^cdefgC^
A10	1.64 ± 0.10^abcA^	29.78 ± 0.05^efB^	72.77 ± 2.64^cdefghC^	0.33 ± 0.03^abcdeA^	0.94 ± 0.10^abcdeB^	2.04 ± 0.38^abcdefgC^	3.61 ± 0.17^abA^	60.50 ± 0.11^fghB^	147.59 ± 5.10^cdefghiC^
A11	1.76 ± 0.10^abcdeA^	31.76 ± 0.20^fghijB^	75.29 ± 0.34^efghC^	0.33 ± 0.08^abcdeA^	0.68 ± 0.05^abcdA^	1.87 ± 0.37^abcdefB^	3.85 ± 0.17^abcA^	64.21 ± 0.37^ghijkB^	152.45 ± 0.60^efghiC^
A12	2.61 ± 0.05^lmnoA^	29.73 ± 0.13^efB^	74.33 ± 2.25^defghC^	0.12 ± 0.03^abcA^	0.45 ± 0.02^abcB^	1.34 ± 0.22^abcdC^	5.35 ± 0.09^fghiA^	59.92 ± 0.24^efB^	150.01 ± 4.40^defghiC^
A13	2.50 ± 0.11^klmnA^	30.59 ± 0.58^efghB^	68.84 ± 1.82^cdefC^	0.02 ± 0.01^aA^	0.19 ± 0.01^aA^	0.89 ± 0.13^aB^	5.02 ± 0.23^efgA^	61.37 ± 1.16^fghB^	138.57 ± 3.59^cdefgC^
A14	2.34 ± 0.11^jklmA^	30.06 ± 0.36^efgB^	65.44 ± 1.53^cdC^	0.03 ± 0.01^aA^	0.32 ± 0.03^abA^	1.12 ± 0.24^abB^	4.69 ± 0.22^cdefgA^	60.44 ± 0.69^fgB^	132.00 ± 3.19^cdC^
A15	1.67 ± 0.04^abcdA^	30.67 ± 0.14^efghiB^	67.09 ± 1.04^cdeC^	0.34 ± 0.03^abcdeA^	0.98 ± 0.09^abcdeB^	1.78 ± 0.36^abcdefC^	3.68 ± 0.11^abA^	62.32 ± 0.33^fghiB^	135.97 ± 2.28^cdeC^
Serbia	2.61 ± 0.51^a^	34.99 ± 5.16^a^	74.33 ± 5.38^a^	0.97 ± 0.49^a^	1.53 ± 0.46^a^	2.64 ± 0.88^a^	6.20 ± 1.22^a^	71.51 ± 10.48^a^	151.30 ± 10.73^a^
Argentina	2.02 ± 0.36^b^	28.44 ± 4.98^b^	68.44 ± 7.24^b^	0.61 ± 0.82^a^	1.20 ± 1.26^a^	1.99 ± 1.08^a^	4.64 ± 1.17^b^	58.08 ± 10.08^b^	138.88 ± 14.75^b^

Values are means ± standard deviation (n = 3).

Different lower-case letters in the same column indicate significantly different values (p < 0.05), according to post hoc Tukey's HSD.

Different upper-case letters in same raw indicate significantly different values of inidividual oxidative parameter (PV, AnV, totox) between days during Schaal oven test (p < 0.05), according to post hoc Tukey's HSD.

CT content indicates oxidation products of gamma-linolenic, arachidonic, eicosapentaenoic, docosahexaenoic fatty acids [91] poorly present in sunflower oil what was a reason of low CT content (less than 1) in the tested samples, even after 8 days of the test exposure. Similar results were obtained by Prescha et al. [92] in the cold pressed safflower oil whose fatty acid composition is similar to sunflower oils tested in this paper (20.6 ± 4.8% oleic acid; 67.3 ± 3.0% linoleic acid). The CT content in the initial safflower sample was 0.65 ± 0.46, after 3 days 0.72 ± 0.61, while after 6 days amounted to 0.82 ± 0.55.

A statistically significant difference (p˂0.05) was observed between samples grown in Serbia and Argentina in CD content on 4th and 8th day of the test exposure, as well as in PV and totox index in the initial samples and during the test. Samples grown in Argentina had significantly higher unsaturated C18:2 fatty acid content (61.98 ± 2.22%) compared to samples grown in Serbia (57.14 ± 2.70%) what potentially was a reason of higher CD content found on 4th day of the test in the Argentina samples. Significantly higher CD content (8th day of the test), as well as PV and totox index (0^th^, 4th and 8th day) in the samples grown in Serbia might be a consequence of the significantly lower total phytosterols, total phenols and total chlorophylls content 1505.82 ± 152.96 mg/kg, 8.96 ± 0.71 mg/kg and 0.73 ± 0.31 mg/kg, respectively compared to samples from Argentina (1582.89 ± 167.17 mg/kg of total phytosterols, 9.89 ± 0.69 mg/kg total phenols and 1.25 ± 0.31 mg/kg total chlorophylls). These compounds, as previously was mentioned, have antioxidative effect.

### 3.4 Principal component analysis

**Fig. 1:**
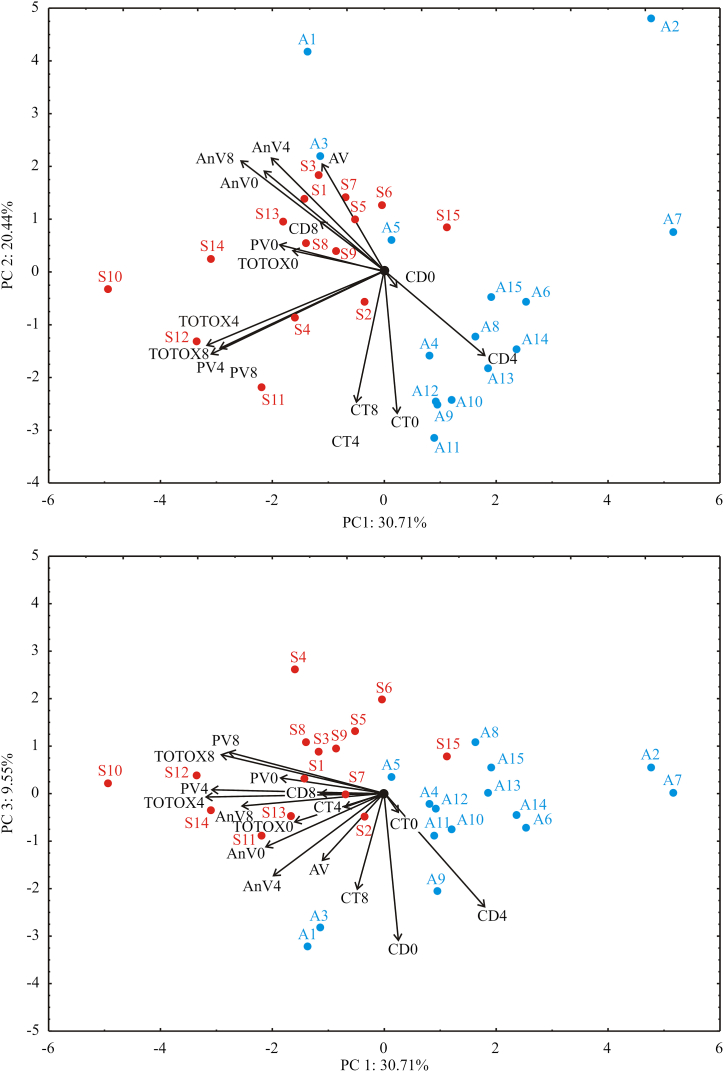
PCA biplot for oil stability parameters of investigated cold pressed oils. PC1, PC2 and PC3: first, second and third principal components; Red labels – sunflower hybrids grown in Serbia: S1 – S15; Blue labels – sunflower hybrids grown in Argentina: A1 – A15; AV: acid value, PV0, PV4 and PV8: peroxide value; AnV0, AnV4 and AnV8: anisidine value; CD0, CD4 and CD8: conjugated dienes; CT0, CT4 and CT8: conjugated trienes and TOTOX0, TOTOX4 and TOTOX8: total oxidation index; 0, 4 and 8: 0^th^, 4th and 8th day accelerated stability test. (For interpretation of the references to color in this figure legend, the reader is referred to the Web version of this article.)

The principal component analysis (PCA) of oil quality characteristics data explained that the first three components accounted for 60.70% of the total variance in the fourteen variables factor space (oil quality characteristics). Considering the map of the PCA performed on the data, the quality parameters: PV4 (14.9% of total variance, based on correlations), PV8 (12.1%), TOTOX4 (15.9%), TOTOX8 (13.4%), AnV0 (7.1%) and AnV8 (10.3%) exhibited negative scores according to first principal component (Fig. 1). The positive influence toward PC2 coordinate was obtained for quality parameters: AV (7.7%), AnV4 (8.4%) and AnV8 (8.1%), whilst the negative influence on PC2 calculation was observed for: CT0 (13.9%), CT4 (17.7) and CT8 (11.8%). The negative influence on PC3 coordinate was observed for: AV (7.2%), AnV4 (10.9%), CT8 (14.7%), CD0 (34.1%) and CD4 (20.7%). Based on the applied PCA analysis, Serbia samples generally had higher values of parameters negatively influential on PC1 (PV4, PV8, TOTOX4, TOTOX8, AnV0 and AnV8), while the reversed situation is observed in the samples from Argentina.

**Fig. 2:**
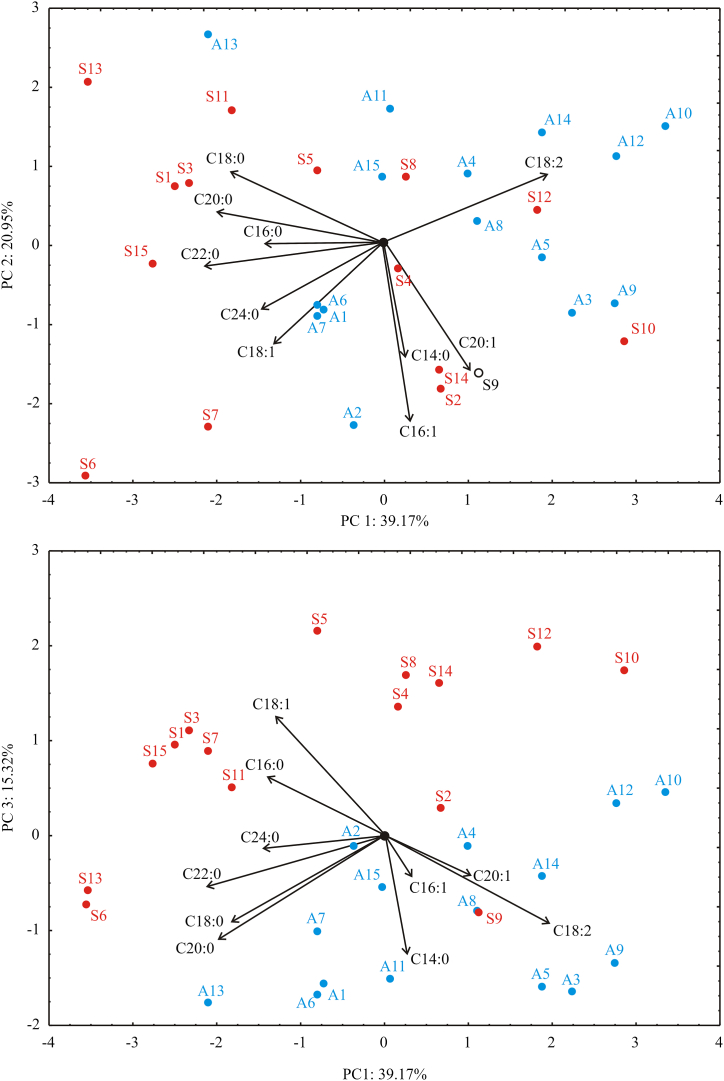
PCA biplot for fatty acids composition of investigated cold pressed oils. PC1, PC2 and PC3: first, second and third principal components; Red labels – sunflower hybrids grown in Serbia: S1 – S15; Blue labels – sunflower hybrids grown in Argentina: A1 – A15; C14:0, C16:0, C16:1, C18:0, C18:1, C18:2, C20:0, C20:1, C22:0 and C24:0: fatty acids. (For interpretation of the references to color in this figure legend, the reader is referred to the Web version of this article.)

The principal component analysis (PCA) of fatty acid profile explained that the first three components accounted for 75.43% of the total variance in the ten variables factor space (fatty acid profile). Considering the map of the PCA performed on the data, the fatty acid profile parameters: C18:2 (16.5% of total variance, based on correlations) showed a positive influence on the first principal component, while C18:0 (15.0%), C20:0 (17.4%), C16:0 (8.7%), C22:0 (20.3%), C24:0 (9.3%) and C18:1 (7.6%) exhibited a negative scores according to first principal component (Fig. 2). The negative influence toward PC2 coordinate was obtained for fatty acid profile parameters: C18:1 (11.4%), C16:1 (36.1%), C14:0 (15.2%) and C20:1 (18.6%). The positive influence on PC3 coordinate was noticed for: C18:1 (22.8%), while the negative influences on PC3 coordinate was observed for: C18:0 (11.5%), C20:0 (16.6%), C14:0 (22.6%) and C18:2 (11.8%). Results showed (Fig. 2) that the distribution of the examined samples to the samples from Serbia and Argentina was based on PC3, and the most effective variables to discriminate the oils were C18:1, C18:2, C18:0, C20:0, and C14:0 fatty acids.

**Fig. 3:**
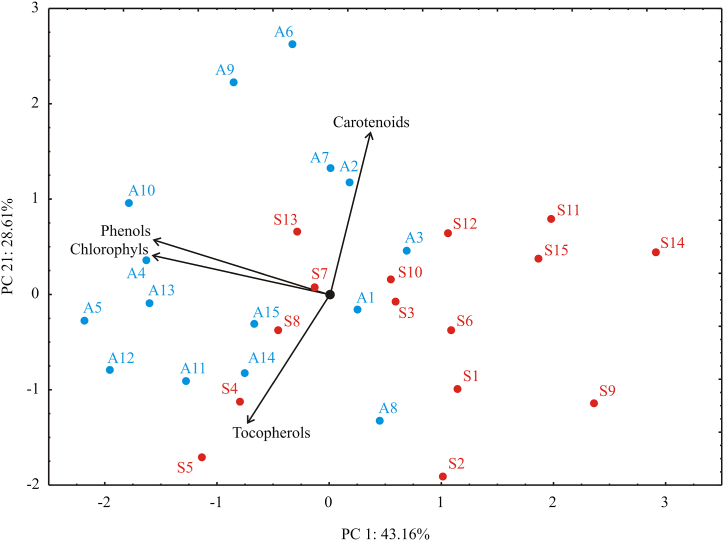
PCA biplot for bioactive compounds of investigated cold pressed oil. PC1 and PC2: first and second principal components; Red labels – sunflower hybrids grown in Serbia: S1 – S15; Blue labels – sunflower hybrids grown in Argentina: A1 – A15. (For interpretation of the references to color in this figure legend, the reader is referred to the Web version of this article.)

The principal component analysis (PCA) of bioactive compounds explained that the first two components accounted for 71.76% of the total variance in the four variables factor space (bioactive compounds). Considering the map of the PCA performed on the data, the bioactive compounds parameters: phenols (44.0%) and chlorophyll (44.1%) exhibited negative score according to first principal component (Fig. 3). The negative influence toward PC2 coordinate was obtained for tocopherols (34.4%), while the positive influence on the second principal component was observed for carotenoids content (55.6%). Fig. 3 indicates a higher total phenols and chlorophyll content found mainly in the samples from Argentina compared to the samples from Serbia.

### 3.5 ANN model

Artificial neural network modeling has previously been used to predict the oxidative stability of other samples. Namely, Barradas Filho et al. [93] used artificial neural network to predict viscosity, iodine value and induction period of biodiesel, while Burgaz et al. [94] predicted thermal stability, crystallinity and thermomechanical properties of poly (ethylene oxide)/clay nanocomposites.

According to ANN performance (sum of *r*^2^ and SOSs for all variables in one ANN), it was noticed that the optimal number of neurons in the hidden layer was 15 (network MLP 14-15-16), when obtaining high values of *r*^2^ (0.997, for training cycle) and also low values of SOS (0.002), Table S1.

The ANN model is complex (481 weights-biases) because of the high nonlinearity of the developed system [70,95].

*SOS* obtained with ANN model were of the same order of magnitude as experimental errors in the literature [96,97].

The quality of the model fit was tested and the residual analysis of the developed model was presented in Table S2.

The ANN model had an insignificant lack of fit tests, which means the model satisfactorily predicted the quality of oil, based on the bioactive compounds and fatty acids profile. A high *r*^*2*^ is indicative that the variation was accounted for and that the data fitted the proposed model satisfactorily [97,98].

The advantages of applying the artificial neural network model in the oil oxidative stability investigation are multifaceted because using the obtained model based on the input parameters (fatty acids composition and bioactive components content) it is possible to avoid long-term investigation of the oxidative stability of oil. The limitation of this analysis is that the model is suitable only for samples with input parameters included in the tested samples. Probably, for the future analysis, it would be necessary to select as many different samples as possible, with a heterogeneous fatty acids composition and bioactive components content, in order to get a model applicable to as many samples as possible. However, future work in this direction requires consideration and breeders who will do selection in the direction of changing the fatty acids composition and bioactive components content.

### 3.6 Sensitivity analysis

**Fig S1.tif:**
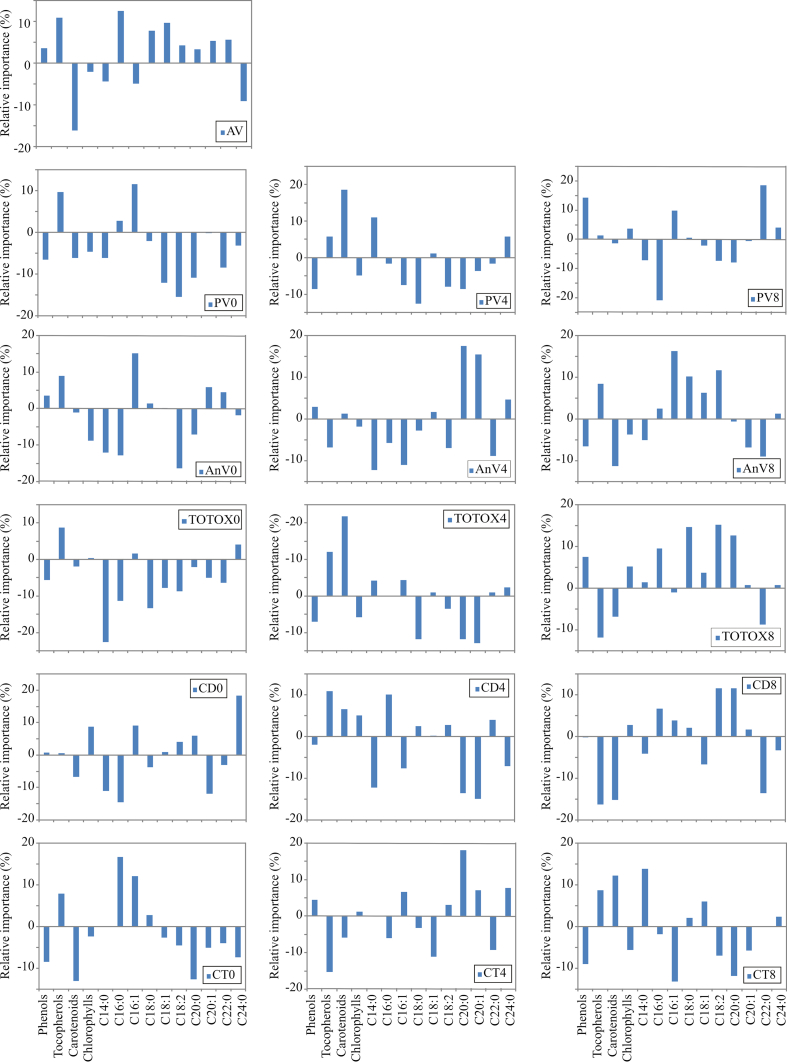


The influence of bioactive compounds and fatty acids profile on oil quality parameters was studied as well (Fig. S1).

Although the bioactive compounds, as already mentioned, contribute to the oxidative stability of the oil, results showed that the content of total phenols, tocopherols, carotenoids, and chlorophyll may affect the increase in the values of parameters. In the initial samples, the negative influence of bioactive compounds on the examined parameters was mainly noticed. However, the results of sensitivity analysis applied in this paper for some parameters on the 4th and 8th day indicate a slightly different situation. This effect of bioactive compounds can be explained by their low concentrations in sunflower oil, therefore the fatty acid composition had a dominant effect on the oxidative stability of the tested oils, as reported previously [1]. The content of C18:2 fatty acid, as the most dominant fatty acid, had a positive effect on AnV8, TOTOX8 and CD8, which indicates that oxidative changes occurred in the linoleic acid after 8 days of exposure. C18:2 is oxidatively the most unstable due to the presence of two double bonds. Other fatty acids present in the oils were saturated and monounsaturated fatty acids, less changed under the test conditions.

## 4 Conclusions

Differences in oxidative characteristics, fatty acids composition and bioactive compounds content between cold pressed oils obtained from same sunflower hybrids grown in Serbia and Argentina were investigated. Applied PCA analysis was demonstrated that samples from Serbia were characterized by a higher content of oleic acid, PV4, PV8, TOTOX4, TOTOX8, AnV0, and AnV8, while samples grown in Argentina were characterized by a higher content of linoleic, stearic, arachidic, and myristic acid, as well as total phenols and chlorophyll content.

This paper also proved a possibility of oxidative stability parameters prediction based on the fatty acid composition and bioactive compounds content. Namely, a new artificial neural network model for oxidative characteristic prediction was developed. Based on the validation model parameters (χ^2^ from 0.000 to 4.228; RMSE from 0.002 to 1.405; MBE from −0.088 to 0.000; MPE from 0.609 to 36.879; *r*^2^ from 0.980 to 0.999), ANN model was adequate to present the experimental results. So, it is possible to predict stability parameters (AV, CD, CT, PV, AnV, TOTOX) of cold pressed sunflower oils obtained from hybrids grown in Serbia and Argentina based on the fatty acid composition and bioactive compounds content (total phenols, tocopherols, carotenoids and chlorophylls).

## Additional information

Supplementary content related to this article has been published online at [URL].

## Author contribution statement

Tanja Lužaić;, PhD: Performed the experiments; Analyzed and interpreted the data; Wrote the paper. Snežana Kravić, PhD; Zorica Stojanović, PhD: Performed the experiments. Nada Grahovac, PhD: Conceived and designed the experiments; Performed the experiments; Contributed reagents, materials, analysis tools or data. Siniša Jocić;, PhD; Sandra Cvejić;, PhD: Conceived and designed the experiments; Contributed reagents, materials, analysis tools or data. Lato Pezo, PhD: Analyzed and interpreted the data; Wrote the paper. Ranko S. Romanić, Ph.D.: Conceived and designed the experiments; Analyzed and interpreted the data; Contributed reagents, materials, analysis tools or data; Wrote the paper.

## Data availability statement

Data included in article/supp. material/referenced in article.

## Declaration of competing interest

The authors declare that they have no known competing financial interests or personal relationships that could have appeared to influence the work reported in this paper.

## Appendix A Supplementary data

Supplementary material.docx Supplementary material.docx

## References

[1] Choe E., Min D.B. (2006). Mechanisms and factors for edible oil oxidation. Compr. Rev. Food Sci. Food Saf..

[2] Redondo-Cuevas L., Castellano G., Torrens F., Raikos V. (2018). Revealing the relationship between vegetable oil composition and oxidative stability: a multifactorial approach. J. Food Compos. Anal..

[3] Lajara J.R., Diaz U., Quidiello R.D. (1990). Definite influence of location and climatic conditions on the fatty acid composition of sunflower seed oil. JAOCS (J. Am. Oil Chem. Soc.).

[4] Saad Bin Mustafa H., Batool N., Iqbal Z., ul Hasan E., Mahmood T. (2015). Effect of fruit position and variable temperature on chemical composition of seeds in Brassica, cotton, sunflower and maize crops. Researcher.

[5] Matsuzaki T., Koiwai A., Iwai S. (1988). Effects of temperature on seed fatty acid composition in ovary culture of tobacco. Agric. Biol. Chem..

[6] Onemli F. (2012). Changes in oil fatty acid composition during seed development of sunflower. Asian J. Plant Sci..

[7] Werteker M., Lorenz A., Johannes H., Berghofer E., Findlay C.S. (2010). Environmental and varietal influences on the fatty acid composition of rapeseed, soybeans and sunflowers. J. Agron Crop Sci..

[8] Fernandez-Martinez J., Jimenez A., Dominguez J., Garcia J.M., Garces R., Mancha M. (1989). Genetic analysis of the high oleic acid content in cultivated sunflower (*Helianthus annuus* L.). Euphytica.

[9] Grunvald A.K., De Carvalho C.G.P., Leite R.S., Mandarino J.M.G., De Bastos Andrade C.A., Amabile R.F., De Paulo Campos Godinho V. (2013). Influence of temperature on the fatty acid composition of the oil from sunflower genotypes grown in tropical regions. J. Am. Oil Chem. Soc..

[10] Naeli M.H., Farmani J., Zargaraan A. (2017). Rheological and physicochemical modification of trans-free blends of palm stearin and soybean oil by chemical interesterification. J. Food Process. Eng..

[11] Smith S.A., King R.E., Min D.B. (2007). Oxidative and thermal stabilities of genetically modified high oleic sunflower oil. Food Chem..

[12] Min D., Boff J. (2002).

[13] Choe E., Min D.B. (2006). Chemistry and reactions of reactive oxygen species in foods. Crit. Rev. Food Sci. Nutr..

[14] Taghvaei M., Jafari S.M. (2015). Application and stability of natural antioxidants in edible oils in order to substitute synthetic additives. J. Food Sci. Technol..

[15] Gramza-Michalowska A., Korczak J., Regula J. (2007). Use of plant extracts in summer and winter season butter oxidative stability improvement. Asia Pac. J. Clin. Nutr..

[16] Kozlowska M., Gruczynska E. (2018). Comparison of the oxidative stability of soybean and sunflower oils enriched with herbal plant extracts. Chem. Pap..

[17] McClements D.J., Decker E.A. (2000). Lipid oxidation in oil-in-water emulsions : impact of molecular environment on chemical. J. Food Sci..

[18] Javidipour I., Erinç H., Basturk A., Tekin A. (2017). Oxidative changes in hazelnut, olive, soybean, and sunflower oils during microwave heating. Int. J. Food Prop..

[19] Grompone M.A. (2020). Bailey's Industrial Oil and Fat Products.

[20] Skoric D., Jocic S., Sakac Z., Lecic N. (2008). Genetic possibilities for altering sunflower oil quality to obtain novel oils. Can. J. Physiol. Pharmacol..

[21] Jocic S., Miladinović D., Kaya Y., Martinez-Force E., Dunford N.T., Salas J.J. (2015). Sunflower Chemistry, Production, Processing, and Utilization.

[22] Melgarejo M. (1998). Girasol en Argentina. Aceites y Grasas.

[23] Gotor A.A., Farkas E., Berger M., Labalette F., Centis S., Daydé J., Calmon A. (2007). Determination of tocopherols and phytosterols in sunflower seeds by NIR spectrometry. Eur. J. Lipid Sci. Technol..

[24] Marmesat S., Velasco L., Ruiz-Mendez M.V., Fernandez-Martinez J.M., Dobarganes C. (2008). Thermostability of genetically modified sunflower oils differing in fatty acid and tocopherol compositions. Eur. J. Lipid Sci. Technol..

[25] Velasco L., Perez-Vich B., Fernandez-Martinez J.M. (2004). Novel variation for the tocopherol profile in a sunflower created by mutagenesis and recombination. Plant Breed..

[26] De Leonardis A., Macciola V., Di Rocco A. (2003). Oxidative stabilization of cold-pressed sunflower oil using phenolic compounds of the same seeds. J. Sci. Food Agric..

[27] Leung J., Fenton T.W., Clandinin D.R. (1981). Phenolic components of sunflower flour. J. Food Sci..

[28] Milic B., Stojanovic S., Vucurevic N., Turcic M. (1968). Chlorogenic and quinic acids in sunflower meal. J. Sci. Food Agric..

[29] Pedrosa M.M., Muzquiz M., Garcia-Vallejo C., Burbano C., Cuadrado C., Ayet G., Robredo L.M. (2000). Determination of caffeic and chlorogenic acids and their derivatives in different sunflower seeds. J. Sci. Food Agric..

[30] Valavanidis A., Nisiotou C., Papageorgiou Y., Kremli I., Satravelas N., Zinieris N., Zygalaki H. (2004). Comparison of the radical scavenging potential of polar and lipidic fractions of olive oil and other vegetable oils under normal conditions and after thermal treatment. J. Agric. Food Chem..

[31] Nyam K.L., Tan C.P., Lai O.M., Long K., Che Man Y.B. (2009). Physicochemical properties and bioactive compounds of selected seed oils. LWT - Food Sci. Technol. (Lebensmittel-Wissenschaft -Technol.).

[32] Kiokias S., Gordon M.H. (2003). Dietary supplementation with a natural carotenoid mixture decreases oxidative stress. Eur. J. Clin. Nutr..

[33] Kiokias S., Oreopoulou V. (2006). Antioxidant properties of natural carotenoid extracts against the AAPH-initiated oxidation of food emulsions. Innovative Food Sci. Emerging Technol..

[34] Palozza P. (1998). Nutrition Reviews.

[35] De Leonardis A., Mcciola V., De Felice M. (2001). Chemical and commercial characteristics of cold pressed sunflower oils. Italian food and beverage technology.

[36] Dimakou C., Oreopoulou V. (2012). Antioxidant activity of carotenoids against the oxidative destabilization of sunflower oil-in-water emulsions. LWT - Food Sci. Technol. (Lebensmittel-Wissenschaft -Technol.).

[37] Tuberoso C.I.G., Kowalczyk A., Sarritzu E., Cabras P. (2007). Determination of antioxidant compounds and antioxidant activity in commercial oilseeds for food use. Food Chem..

[38] Kiokias S., Varzakas T., Oreopoulou V. (2008). Critical Reviews in Food Science and Nutrition.

[39] Kiokias S., Gordon M.H. (2004). Antioxidant properties of carotenoids in vitro and in vivo. Food Rev. Int..

[40] Dimic E., Premovic T., Radocaj O., Vujasinovic V., Takaci A. (2018). Influence of seed quality and storage time on the characteristics of cold-pressed sunflower oil: impact on bioactive compounds and colour. Riv. Ital. Sostanze Grasse.

[41] Martinez M.L., Penci M.C., Ixtaina V., Ribotta P.D., Maestri D. (2013). Effect of natural and synthetic antioxidants on the oxidative stability of walnut oil under different storage conditions. LWT - Food Sci. Technol. (Lebensmittel-Wissenschaft -Technol.).

[42] Lutterodt H., Slavin M., Whent M., Turner E., Yu L. (2011). Fatty acid composition, oxidative stability, antioxidant and antiproliferative properties of selected cold-pressed grape seed oils and flours. Food Chem..

[43] Parry J., Su L., Luther M., Zhou K., Peter Yurawecz M., Whittaker P., Yu L. (2005). Fatty acid composition and antioxidant properties of cold-pressed marionberry, boysenberry, red raspberry, and blueberry seed oils. J. Agric. Food Chem..

[44] Velasco J., Dobarganes C. (2002). Oxidative stability of virgin olive oil. Eur. J. Lipid Sci. Technol..

[45] Berger A., Jones P.J.H., Abumweis S.S. (2004). Lipids in Health and Disease.

[46] Winkler J.K., Warner K. (2008). The effect of phytosterol concentration on oxidative stability and thermal polymerization of heated oils. Eur. J. Lipid Sci. Technol..

[47] Vlahakis C., Hazebroek J. (2000). Phytosterol accumulation in canola, sunflower, and soybean oils: effects of genetics, planting location, and temperature. JAOCS (J. Am. Oil Chem. Soc.).

[48] Lužaić T., Romanić R., Grahovac N., Jocić S., Cvejić S., Hladni N., Pezo L. (2021). Prediction of mechanical extraction oil yield of new sunflower hybrids: artificial neural network model. J. Sci. Food Agric..

[49] Luzaic T., Grahovac N., Hladni N., Romanic R. (2022). Food Science and Technology.

[50] ISO 12966-4 (2015).

[51] ISO 12966-2 (2017).

[52] ISO 9936 (2016).

[53] Haiyan Z., Bedgood D.R., Bishop A.G., Prenzler P.D., Robards K. (2007). Endogenous biophenol, fatty acid and volatile profiles of selected oils. Food Chem..

[54] (1977). Methods of Analysis of Fats and Oils. Other Methods. Determination of Carotene in Vegetable Oils.

[55] Pokorny J., Kalinova L., Dysseler P. (1995). Determination of chlorophyll pigments in crude vegetable oils. Pure Appl. Chem..

[56] Araujo L.B.D.C., Silva S.L., Galvao M.A.M., Ferreira M.R.A., Araujo E.L., Randau K.P., Soares L.A.L. (2013). Total phytosterol content in drug materials and extracts from roots of *Acanthospermum hispidum* by UV-VIS spectrophotometry. Revista Brasileira de Farmacognosia.

[57] Gomes T., Caponio F., Bruno G., Summo C., Paradiso V.M. (2010). Effects of monoacylglycerols on the oxidative stability of olive oil. J. Sci. Food Agric..

[58] Maszewska M., Florowska A., Dluzewska E., Wroniak M., Marciniak-Lukasiak K., Zbikowska A. (2018). Oxidative stability of selected edible oils. Molecules.

[59] Naderi M., Farmani J., Rashidi L. (2018). The impact of saturated monoacylglycerols on the oxidative stability of Canola oil under various time/temperature conditions. Grasas Aceites.

[60] ISO 3960 (2017).

[61] ISO 6885 (2016).

[62] ISO 3656:2013/Amd. 1:2017. Animal and Vegetable Fats and Oils - Determination of Ultraviolet Absorbance Expressed as Specific UV Extinction - Amendment 1. International Organization for Standardization, Geneva, Switzerland.

[63] Oomah B.D., Ladet S., Godfrey D.V., Liang J., Girard B. (2000). Characteristics of raspberry (*Rubus idaeus* L.) seed oil. Food Chem..

[64] Wai W.T., Saad B., Lim B.P. (2009). Determination of TOTOX value in palm oleins using a FI-potentiometric analyzer. Food Chem..

[65] ISO 660 (2009).

[66] Taylor B.J. (2006). Methods and Procedures for the Verification and Validation of Artificial Neural Networks.

[67] Basheer I.A., Hajmeer M. (2000). Artificial neural networks: fundamentals, computing, design, and application. J. Microbiol. Methods.

[68] Pezo L., Curcic B., Filipovic V., Nicetin M., Koprivica G., Misljenovic N., Levic L. (2013). Artificial neural network model of pork meat cubes osmotic dehydratation. Hem. Ind..

[69] StatSoft, Inc. (2010). http://www.statsoft.com/.

[70] Kollo T., von Rosen D. (2005). Advanced Multivariate Statistics with Matrices.

[71] Yoon Y., Swales G., Margavio T.M. (2017). A comparison of discriminant analysis versus artificial neural networks. J. Oper. Res. Soc..

[72] Arsenović M., Pezo L., Stanković S., Radojević Z. (2015). Factor space differentiation of brick clays according to mineral content: prediction of final brick product quality. Appl. Clay Sci..

[73] Alimentarius Codex (1999).

[74] Gliszczynska-Swiglo A., Sikorska E., Khmelinskii I.V., Sikorski M. (2007). Tocopherol content in edible plant oils. Pol. J. Food Nutr. Sci..

[75] Okogeri O., Tasioula-Margari M. (2002). Changes occurring in phenolic compounds and α-tocopherol of virgin olive oil during storage. J. Agric. Food Chem..

[76] Vissers M.N., Zock P.L., Katan M.B. (2004). Bioavailability and antioxidant effects of olive oil phenols in humans: a review. Eur. J. Clin. Nutr..

[77] Boskou D. (2006). http://www.ncbi.nlm.nih.gov/pubmed/18598039.

[78] Parker T.D., Adams D.A., Zhou K., Harris M., Yu L. (2003). Fatty acid composition and oxidative stability of cold-pressed edible seed oils. J. Food Sci..

[79] Matthaus B., Bruhl L. (2004). Cold-pressed edible rapeseed oil production in Germany. INFORM - International News on Fats, Oils and Related Materials.

[80] Pokorny J., Velisek J., Panek J., Kanova J., Parizkova H., Holasova M., Koplik R., Cmolik J. (1993). Minor lipophilic components in crude rapeseed oil. Potravinarske Vedy - UZPI.

[81] Karabagias I., Michos C., Badeka A., Kontakos S., Stratis I., Kontominas M.G. (2013). Classification of Western Greek virgin olive oils according to geographical origin based on chromatographic, spectroscopic, conventional and chemometric analyses. Food Res. Int..

[82] Rodriguez-Amaya D.B. (2010). Quantitative analysis, in vitro assessment of bioavailability and antioxidant activity of food carotenoids-A review. J. Food Compos. Anal..

[83] Lanfer-Marquez U.M., Barros R.M.C., Sinnecker P. (2005). Antioxidant activity of chlorophylls and their derivatives. Food Res. Int..

[84] Cercaci L., Passalacqua G., Poerio A., Rodriguez-Estrada M.T., Lercker G. (2007). Composition of total sterols (4-desmethyl-sterols) in extravirgin olive oils obtained with different extraction technologies and their influence on the oil oxidative stability. Food Chem..

[85] Ayerdi Gotor A., Farkas E., Berger M., Labalette F., Centis S., Dayde J., Calmon A. (2007). Determination of tocopherols and phytosterols in sunflower seeds by NIR spectrometry. Eur. J. Lipid Sci. Technol..

[86] Piironen V., Lindsay D.G., Miettinen T.A., Toivo J., Lampi A.M. (2000). Plant sterols: biosynthesis, biological function and their importance to human nutrition. J. Sci. Food Agric..

[87] Konuskan D.B., Arslan M., Oksuz A. (2019). Physicochemical properties of cold pressed sunflower, peanut, rapeseed, mustard and olive oils grown in the Eastern Mediterranean region. Saudi J. Biol. Sci..

[88] Mehta B.M., Kumar Jain A., Darji V.B., Aparnathi K.D. (2018). Evaluation of different methods to monitor primary stage of oxidation of heat clarified milk fat (ghee). J. Food Process. Preserv..

[89] Shantha N.C., Decker E.A. (1994). Rapid, sensitive, iron-based spectrophotometric methods for determination of peroxide values of food lipids. J. AOAC Int..

[90] Shahidi F., Wanasundara U. (2002).

[91] Takagi T., Wakasa N., Miyashita K. (1987). Formation of conjugated diene and triene products in lipoxygenase oxidation of C18, C20, C22 PUFAs. JAOCS (J. Am. Oil Chem. Soc.).

[92] Prescha A., Grajzer M., Dedyk M., Grajeta H. (2014). The antioxidant activity and oxidative stability of cold-pressed oils. J. Am. Oil Chem. Soc..

[93] Barradas Filho A.O., Barros A.K.D., Labidi S., Viegas I.M.A., Marques D.B., Romariz A.R.S., Marques E.P. (2015). Application of artificial neural networks to predict viscosity, iodine value and induction period of biodiesel focused on the study of oxidative stability. Fuel.

[94] Burgaz E., Yazici M., Kapusuz M., Alisir S.H., Ozcan H. (2014). Prediction of thermal stability, crystallinity and thermomechanical properties of poly(ethylene oxide)/clay nanocomposites with artificial neural networks. Thermochim. Acta.

[95] Montgomery D.C. (1984).

[96] Madamba P.S. (2002). The response surface methodology: an application to optimize dehydration operations of selected agricultural crops. LWT - Food Sci. Technol. (Lebensmittel-Wissenschaft -Technol.).

[97] Turanyi T., Tomlin A.S. (2014).

[98] Valous N.A., Mendoza F., Sun D.W. (2010). Emerging non-contact imaging, spectroscopic and colorimetric technologies for quality evaluation and control of hams: a review. Trends Food Sci. Technol..

