# Supplementary material for: Investigation of oxidative characteristics, fatty acid composition and bioactive compounds content in cold pressed oils of sunflower grown in Serbia and Argentina
Source: Heliyon. 2023 Jul 15;9(7):e18201. doi: 10.1016/j.heliyon.2023.e18201 (PMC10372673; doi:10.1016/j.heliyon.2023.e18201)
Supplement: Supplementary material.docx [file mmc1.docx]

**SUPPLEMENATARY MATERIAL**

^a^ Faculty of Technology Novi Sad, University of Novi Sad, Bulevar cara Lazara 1, 21000 Novi Sad, Serbia

^b^ Institute of Field and Vegetable Crops, National Institute of the Republic of Serbia, Maksima Gorkog 30, 21000 Novi Sad, Serbia

^c^ Institute of General and Physical Chemistry, University of Belgrade, Studentski trg 12/V, 11000 Belgrade, Serbia

***Corresponding author**: Ranko Romanić; e-mail: [rankor@uns.ac.rs](mailto:rankor@uns.ac.rs)

**Table S1.** ANN summary or observed results.

| Network  name | Performance | | | Error | | | Training  algorithm | Error  function | Hidden  activation | Output  activation |
| --- | --- | --- | --- | --- | --- | --- | --- | --- | --- | --- |
|  | Train. | Test. | Valid. | Train. | Test. | Valid. |  |  |  |  |
| MLP 2-8-43 | 0.997 | 0.936 | 0.982 | 0.002 | 0.021 | 0.010 | BFGS 3311 | SOS | Logistic | Exponential |

**Table S2.** The "goodness of fit" tests for the developed ANN model.

|  | χ^2^ | RMSE | MBE | MPE | *r*^2^ | Skew | Kurt | Mean | StDev | Var |
| --- | --- | --- | --- | --- | --- | --- | --- | --- | --- | --- |
| AV | 0.000 | 0.008 | -0.001 | 1.048 | 0.996 | 0.797 | 2.145 | -0.001 | 0.009 | 0.000 |
| PV0 | 0.004 | 0.041 | -0.009 | 1.281 | 0.995 | -2.008 | 4.974 | -0.009 | 0.041 | 0.002 |
| PV4 | 1.035 | 0.695 | -0.018 | 1.873 | 0.986 | 0.030 | 0.744 | -0.018 | 0.707 | 0.500 |
| PV8 | 0.725 | 0.582 | -0.039 | 0.622 | 0.993 | -0.280 | 0.973 | -0.039 | 0.590 | 0.349 |
| AnV0 | 0.009 | 0.065 | -0.004 | 36.879 | 0.991 | 0.484 | 3.227 | -0.004 | 0.066 | 0.004 |
| AnV4 | 0.009 | 0.064 | -0.008 | 8.421 | 0.995 | -0.845 | 0.232 | -0.008 | 0.065 | 0.004 |
| AnV8 | 0.013 | 0.078 | -0.009 | 10.587 | 0.996 | -0.753 | 1.087 | -0.009 | 0.079 | 0.006 |
| TOTOX0 | 0.000 | 0.008 | -0.002 | 6.077 | 0.998 | -1.730 | 4.929 | -0.002 | 0.008 | 0.000 |
| TOTOX4 | 4.228 | 1.405 | -0.046 | 1.823 | 0.986 | -0.139 | 0.841 | -0.046 | 1.428 | 2.039 |
| TOTOX8 | 2.940 | 1.171 | -0.088 | 0.609 | 0.993 | -0.825 | 1.611 | -0.088 | 1.188 | 1.411 |
| CD0 | 0.000 | 0.002 | 0.000 | 11.503 | 0.997 | -1.205 | 2.418 | 0.000 | 0.002 | 0.000 |
| CD4 | 0.007 | 0.057 | -0.006 | 0.943 | 0.999 | 0.235 | -1.051 | -0.006 | 0.057 | 0.003 |
| CD8 | 0.515 | 0.490 | -0.037 | 4.268 | 0.980 | -0.627 | 1.954 | -0.037 | 0.497 | 0.247 |
| CT0 | 0.000 | 0.004 | -0.001 | 1.407 | 0.998 | -2.744 | 10.246 | -0.001 | 0.004 | 0.000 |
| CT4 | 0.000 | 0.004 | 0.000 | 0.951 | 0.998 | -1.586 | 4.876 | 0.000 | 0.004 | 0.000 |
| CT8 | 0.000 | 0.007 | -0.001 | 0.910 | 0.998 | -1.086 | 3.506 | -0.001 | 0.007 | 0.000 |

χ^2^ - reduced chi-square, MBE - mean bias error, RMSE - root mean square error, MPE - mean percentage error, SSE - sum of squared errors, AARD - average absolute relative deviation (AARD), *r*^2^ - coefficient of determination, Skew - skeweness, Kurt - kurtosis, Mean - mean of the residuals, StDev - standard deviation of residuals, Var - variation of residuals

**Figure captions**

**Figure S1.** The influence of bioactive compounds content (phenols, tocopherols, carotenoids and chlorophylls) and fatty acid (C14:0, C16:0, C16:1, C18:0, C18:1, C18:2, C20:0, C20:1, C22:0 and C24:0) composition of cold pressed oils of sunflower hybrids grown in Serbia and Argentina on its oxidative characteristics (acid value-AV, peroxide value-PV0, PV4, PV8, anisidine value-AnV0, AnV4, AnV8, total oxidation value-TOTOX0, TOTOX4, TOTOX8, conjugated dienes content-CD0, CD4, CD8, conjugated trienes content-CT0, CT4, CT8, during Schaal oven test (0^th^, 4^th^ and 8^th^ day of the test exposure)).
